# Mnn10 Maintains Pathogenicity in *Candida albicans* by Extending α-1,6-Mannose Backbone to Evade Host Dectin-1 Mediated Antifungal Immunity

**DOI:** 10.1371/journal.ppat.1005617

**Published:** 2016-05-04

**Authors:** Shi Qun Zhang, Zui Zou, Hui Shen, Shuai Shuai Shen, Qi Miao, Xin Huang, Wei Liu, Li Ping Li, Si Min Chen, Lan Yan, Jun Dong Zhang, Jing Jun Zhao, Guo Tong Xu, Mao Mao An, Yuan Ying Jiang

**Affiliations:** 1 Shanghai Tenth People's Hospital, and Department of Pharmacology, Tongji University School of Medicine, Shanghai, PR China; 2 Department of Anesthesiology, Changzheng Hospital, Second Military Medical University, Shanghai, PR China; 3 Department of Laboratory Diagnosis, Changhai Hospital, Second Military Medical University, Shanghai, PR China; 4 Department of Dermatology, Shanghai Tongji Hospital, Tongji University School of Medicine, Shanghai, PR China; 5 R&D Center of New Drug, School of Pharmacy, Second Military Medical University, Shanghai, PR China; Geisel School of Medicine at Dartmouth, UNITED STATES

## Abstract

The cell wall is a dynamic structure that is important for the pathogenicity of *Candida albicans*. Mannan, which is located in the outermost layer of the cell wall, has been shown to contribute to the pathogenesis of *C*. *albicans*, however, the molecular mechanism by which this occurs remains unclear. Here we identified a novel α-1,6-mannosyltransferase encoded by *MNN10* in *C*. *albicans*. We found that Mnn10 is required for cell wall α-1,6-mannose backbone biosynthesis and polysaccharides organization. Deletion of *MNN10* resulted in significant attenuation of the pathogenesis of *C*. *albicans* in a murine systemic candidiasis model. Inhibition of α-1,6-mannose backbone extension did not, however, impact the invasive ability of *C*. *albicans in vitro*. Notably, *mnn10* mutant restored the invasive capacity in athymic nude mice, which further supports the notion of an enhanced host antifungal defense related to this backbone change. *Mnn10* mutant induced enhanced Th1 and Th17 cell mediated antifungal immunity, and resulted in enhanced recruitment of neutrophils and monocytes for pathogen clearance *in vivo*. We also demonstrated that *MNN10* could unmask the surface β-(1,3)-glucan, a crucial pathogen-associated molecular pattern (PAMP) of *C*. *albicans* recognized by host Dectin-1. Our results demonstrate that *mnn10* mutant could stimulate an enhanced Dectin-1 dependent immune response of macrophages *in vitro*, including the activation of nuclear factor-κB, mitogen-activated protein kinase pathways, and secretion of specific cytokines such as TNF-α, IL-6, IL-1β and IL-12p40. In summary, our study indicated that α-1,6-mannose backbone is critical for the pathogenesis of *C*. *albicans* via shielding β-glucan from recognition by host Dectin-1 mediated immune recognition. Moreover, our work suggests that inhibition of α-1,6-mannose extension by Mnn10 may represent a novel modality to reduce the pathogenicity of *C*. *albicans*.

## Author Summary

Mannan plays a crucial role in cell wall structure and virulence of the opportunistic pathogen *Candida albicans*. Both the invasive ability of the pathogen and the host defense against the pathogen contribute to the outcome of invasive infection. In the present study, we identified a novel α-1,6-mannosyltransferase, which was responsible for cell wall α-1,6-mannose backbone extension in *C*. *albicans*. We determined that α-1,6-mannose backbone is necessary for the pathogenesis of *C*. *albicans* due to its ability to shield β-(1,3)-glucan from the host Dectin-1 recognition and Th1/Th7 response. Our study highlights a novel strategy to enhance the host immune response towards *C*. *albicans*.

## Introduction

*Candida albicans* is a common fungal microorganism that colonizes the oral, genital and gastrointestinal surfaces of most healthy individuals. The maintenance of colonization is the result of a complex balance between fungal proliferation and host immune recognition. Despite host immune defenses aimed at clearing pathogens, *C*. *albicans* has developed numerous strategies to evade host immune detection [1]. In immunocompromised patients, *C*. *albicans* may disseminate into bloodstream, causing life-threatening systemic candidiasis [2, 3]. The associated mortality rates of systemic infection are reported to be greater than 30%, highlighting the potential critical impact of *C*. *albicans* on global health burden [4–6].

The mature cell wall of *C*. *albicans* is a complex structure of cross-linked polysaccharides and glycosylated proteins. The cell wall is not only required for maintaining cell shape and stability, but also is critically related to immunogenicity and virulence of *C*. *albicans*. The outer layer of the cell wall is comprised of glycosylated mannoproteins that are post-translationally modified by *N*- and *O*-linked mannosides [7]. *N*-linked mannan chains are specifically required for cell morphology, phagocytosis, and immune recognition of *C*. *albicans* by host dendritic cells [8]. The core structure of *N*-mannan is a dolichol pyrophosphate anchored oligosaccharide comprised of three glucose, nine mannose and two *N*-acetylglucosamine residues (Glc_3_Man_9_GlcNAc_2_). The outer chain branched mannan is attached to the *N*-mannan core through an α-1,6-backbone. Addition of the first α-1,6-mannose is catalyzed by mannosyltransferase Och1. Notably, Och1 mutant strains of *C*. *albicans* demonstrate attenuated virulence in animal models with systemic infection [9]. Extension of α-1,6-mannose backbone by mannose residues is performed by the enzyme complexes mannan polymerase I (M-Pol I) and II (M-Pol II) [10]. The α-1,6-backbone is then further modified with additional α-1,2-mannose units by Mnn2 family and Mnn5, which similarly, are critical for *C*. *albicans* virulence in mice or *Galleria mellonella* [11, 12]. The outer side chains are further capped with either α-1,3-mannose or β-1,2-mannose units via Mnn1 family and β-1,2-mannosyltransferases (BMTs). The *C*. *albicans MNN1* gene family contains six members, of which only *MNN14* represent a critical factor for pathogenicity *in vivo* [13]. Bmt1 and Bmt3, which are required for the addition of the first and second β-1,2-mannose units respectively, are not associated with the virulence of *C*. *albicans* [14]. Although a variety of *C*. *albicans* mannosylation mutants have been found to be less pathogenic *in vivo*, the mechanisms of host clearance associated with abnormal mannan structures remains unclear.

The cell wall polysaccharides of *C*. *albicans* are mainly composed of multiple layers of carbohydrates, including mannans, β-glucans, and chitins [3]. These polysaccharides serve as pathogen-associated molecular patterns (PAMPs) that can be recognized by host-expressed pattern recognition receptors (PRRs) to initiate an innate immune response [1]. Several PRRs, such as toll-like receptors (TLRs), spleen tyrosine kinase (Syk)-coupled C-type lectin receptors (CLRs), and nucleotide binding oligomerization domain (Nod)-like receptors (NLRs), can recognize PAMPs on the surface of *C*. *albicans* [15–17]. The PRRs engagement by *C*. *albicans* PAMPs triggers innate immune cells to respond and renders antigen-presenting cells competent to prime T cells. A complex signaling cascades, including nuclear factor-κB (NF-κB) and mitogen-activated protein kinase (MAPK) pathways, among others, lead to Th1 and Th17 activation and an adaptive immune response [18–21].

Dectin-1, a myeloid-expressed Syk-coupled receptor, can recognize β-(1,3)-glucan carbohydrates on the surface of various fungi [22–24]. Clinical studies have demonstrated that patients with Dectin-1 Y238X mutation are highly susceptible to mucosal *C*. *albicans* infection [25]. However, live *C*. *albicans*, including yeast and hyphae forms, binds to Dectin-1 in highly specialized pattern *in vitro*, except in the region between the parental yeast cell and the mature bud [26]. During infection, β-(1,3)-glucan of *C*. *albicans* is completely masked in earlier stages, while large percentages are exposed at later stages in a morphotype-independent fashion [27]. Shielding of β-(1,3)-glucan favors fungal survival and persistence by escaping Dectin-1 mediated immune recognition [28]. Previous studies have indicated that unmasking *C*. *albicans* β-(1,3)-glucan elicits a stronger host immune response towards *C*. *albicans* via several experimental manipulations such as drug treatment and several key genes deletion [29–31].

Mnn10, an important subunit of *cis* Golgi mannan polymerase, was identified as an α-1,6-mannosyltransferase which is responsible for mannan backbone extension in non-pathogenic fungal species such as *Saccharomyces cerevisiae* and *Kluyveromyces lactis* [32, 33]. In the present study, we first characterized the role of α-1,6-mannose backbone in *C*. *albicans* pathogenicity. We demonstrated that inhibition of α-1,6-mannose backbone extension can block the development of invasive *C*. *albicans* infection, and suggested α-1,6-mannose backbone extension is essential for the evasion of host Dectin-1 mediated immune response towards *C*. *albicans*.

## Results

### Mnn10 possesses α-1,6-mannosyltransferase activity, and is required for α-1,6-mannose backbone extension in *C*. *albicans*

**Fig 1:**
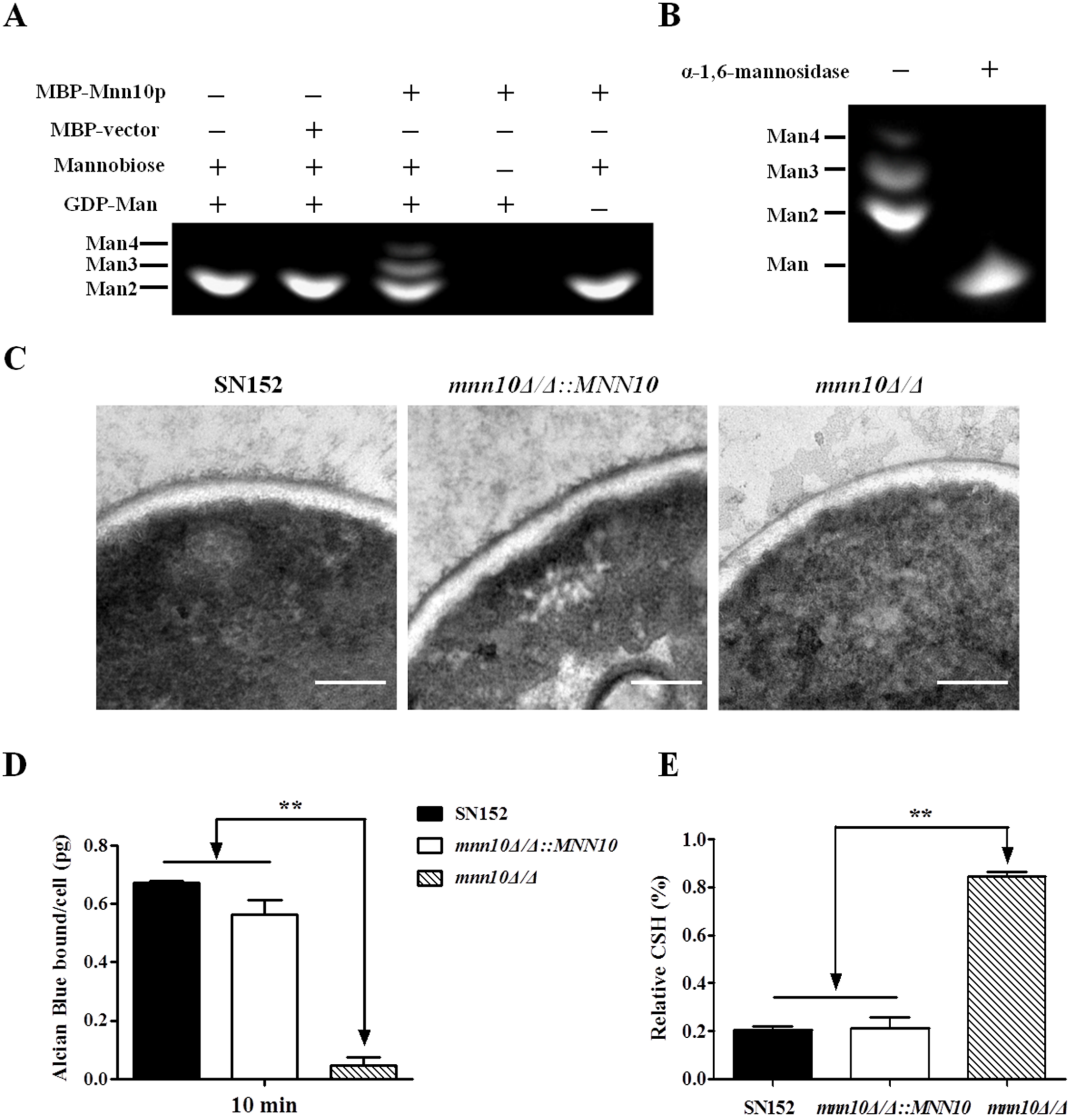
Mnn10 has mannosyltransferase activity and is required for α-1,6-mannose backbone length. (A) Mannosyltransferase activity assay of Mnn10. The reaction of expressed MBP-fused Mnn10 protein or MBP protein incubated with α-1,6-mannobiose (Man 2), GDP-mannose (GDP-Man) or controls. The reaction products were labeled with ANTS and separated by fluorophore-assisted carbohydrate gel electrophoresis (FACE). Man 3, mannotriose; Man 4, mannotetraose. (B) α-1,6-mannose assay. The reaction products of (A) were treated with or without α-1,6-mannosidase treatment, and then subjected to FACE. (C) Representative cell wall ultrastructures of SN152, *mnn10Δ/Δ*::*MNN10* and *mnn10Δ/Δ* strains were observed by transmission electron microscopy. The scale bar represents 0.2 μm. (D) Alcian Blue binding assay. The cells were incubated with Alcian Blue for 10 min and the amount of dye bound to the cell wall were calculated. Data represent the mean amount of dye bound per cell ± SD from triplicates of one representative experiment of three. (E) Cell surface hydrophobicity of the indicated *C*. *albicans* strains was measured by water-hydrocarbon two-phase assay. Data are means ± SD of triplicates of one representative experiment of three. **, *P* < 0.01 [One-way ANOVA with Bonferroni post-test (D, E)].

To analyze enzymatic activity, we used a bacterial expression system to produce MBP-fused Mnn10 protein from a pMAL-p5X vector and purified the protein by amylose magnetic beads (S1 Fig). The purified proteins were subjected to a mannosyltransferase assay system, which required α-1,6-linked mannobiose as an acceptor and GDP-mannose as a donor for proper mannosyltransferase activity [12, 34]. Our results indicated that Mnn10 catalyzes the transformation of GDP-mannose to α-1,6-mannobiose to form mannotriose and mannotetraose (Fig 1A). Consistently, the reaction products could be digested to mannose via α-1,6-mannosidase treatment (Fig 1B). Thus our results demonstrate that Mnn10 is able to transfer mannose from the donor onto the acceptor substrates to form α-1,6-linked oligomannose.

To further confirm the role of *MNN10* in α-1,6-mannose backbone extension, we generated *mnn10Δ/Δ* null mutant strain and *mnn10Δ/Δ*::*MNN10* revertant strain using the homologous recombination method. The genotype was confirmed by PCR and the expression of *MNN10* was determined by quantitative RT-PCR (S2 Fig). When compared to the parental strain SN152, transmission electron microscopy (TEM) analysis noted a significantly shortened external layer of mannan fibril surrounding the cell wall in *mnn10Δ/Δ*. Notably, *MNN10* gene rescue was sufficient to restore the expected length of mannan fibril (Fig 1C).

The phosphomannan of *C*. *albicans* cell wall, characterized by Alcian Blue dye binding, is attached to the branched mannan from the α-1,6-mannose backbone [35]. Therefore, the content of phosphomannan reveals the length of α-1,6-mannose backbone. The Alcian Blue assay demonstrated significantly decreased binding of the dye to *mnn10Δ/Δ* as compared to the parental strain, which could be rescued by reintegration of *MNN10* into *mnn10Δ/Δ*, confirming the role of *MNN10* in α-1,6-mannose backbone extension (Fig 1D). Inhibition of α-1,6-mannose backbone extension via deletion of *MNN10* also affected the surface property of *C*. *albicans* such as hydrophobicity (Fig 1E). Furthermore, the change of cell wall structures impaired the resistance of *mnn10Δ/Δ* to various stresses including calcofluor white, fluconazole, miconazole, and caspofungin (S3 Fig).

Taken together, our results suggest that Mnn10 protein possesses α-1,6-mannosyltransferase activity and is crucial in α-1,6-mannose backbone extension in *C*. *albicans*.

### *MNN10* is required for cell wall polysaccharides biosynthesis and organization in *C*. *albicans*

**Fig 2:**
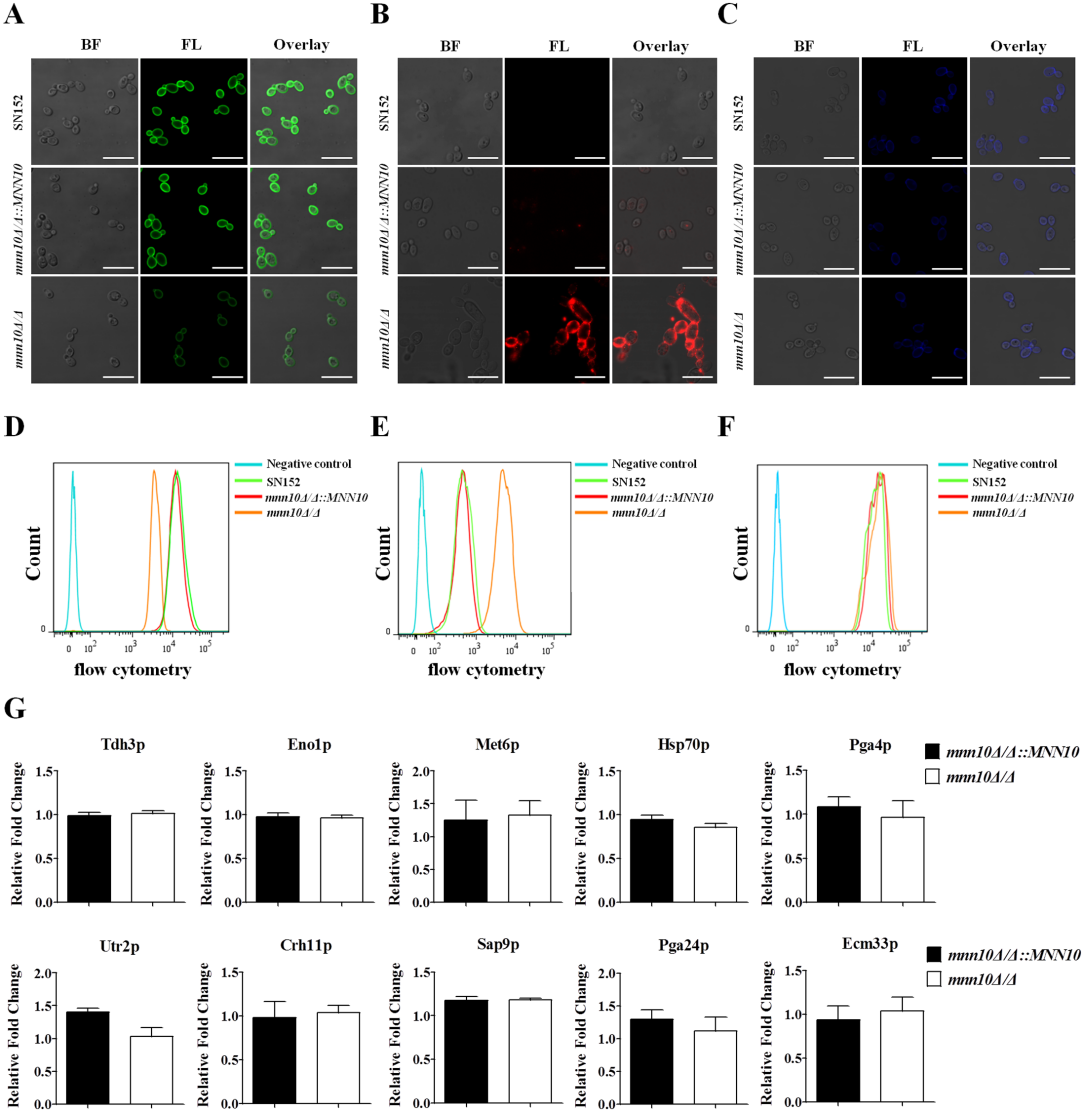
Mnn10 is required for cell wall polysaccharides organization, but not virulence factors attachment. Representative fluorescence micrographs of three cell wall carbohydrate layers from SN152, *mnn10Δ/Δ*::*MNN10* and *mnn10Δ/Δ*, which were stained with ConA-FITC to visualise mannan (A), β-glucan antibody to visualize β-(1,3)-glucan (B) and calcofluor white to visualise chitin (C). Bright field (BF), fluorescence (FL), and overlay are shown individually. Scale bar represents 10 μm. The fluorescence intensity was quantified by flow cytometry [mannan (D), β-(1,3)-glucan (E), chitin (F)]. Data are representative of three independent experiments. (G) Relative fold change of representative virulence related cell wall proteins (CWPs) in *mnn10* mutant compared to *mnn10Δ/Δ*::*MNN10*. The cell wall proteins of SN152, *mnn10Δ/Δ*::*MNN10*, and *mnn10Δ/Δ* strains were analyzed by LC-MS/MS on high-resolution instruments, respectively. Raw files were processed by Max Quant for peptide/protein identification and quantification. The relative data were changed to the ratio of *mnn10Δ/Δ*::*MNN10* and *mnn10Δ/Δ* to SN152. Data are represented as means ± SEM of three independent experiments. *P* > 0.05 (Student’s *t*-test).

The cell wall polysaccharides of *C*. *albicans* consist of an inner layer of chitin and β-glucan, and an outer fibrillar layer of mannan. To examine the effects of *MNN10* deletion on cell wall polysaccharides biosynthesis and organization, *C*. *albicans* yeasts or hyphae were stained with Concanavalin A (ConA), anti-β-glucan antibody and calcofluor white (CFW). We observed and quantified the polysaccharides by confocal laser scanning microscopy and flow cytometry, respectively. ConA staining indicated that *mnn10* mutant, either in yeast cells or hyphae, had a markedly decreased fluorescence intensity which is suggested lower mannose content as compared to the parental or revertant strain (Figs 2A, 2D and S4A). β-(1,3)-glucan, a well-characterized PAMP of *C*. *albicans*, is buried underneath the outer layer of the cell wall. We found a remarkable exposure of β-(1,3)-glucan on the cell surface of *mnn10* mutant (Figs 2B, 2E and S4B). However, no significant difference of CFW binding was visualized in *mnn10* mutant strain, suggesting normal chitin content (Fig 2C and 2F).

The highly glycosylated cell wall proteins (CWPs) of *C*. *albicans* often act as virulence factors and contribute to cell wall integrity, promote biofilm formation, mediate adherence to host cells, and promote invasion of epithelial layers [36–38]. To further evaluate the effect of α-1,6-mannose extension in *C*. *albicans* CWPs anchorage, we analyzed CWPs extraction by LC-MS/MS. Twenty representative CWPs were identified in the cell wall of the parental and *mnn10* mutant strain (S1 Table). We found that *MNN10* deletion had almost no effect on the anchorage of CWPs. Notably several of the proteins are considered to mediate specific roles in the development of invasion, such as cell-surface antigens involved in virulence (Tdh3p, Eno1p, Met6p, Hsp70p), proteins involved in cell wall biosynthesis and assembly (Pga4p, Utr2p, Crh11p), and proteins involved in adhesions and cell wall morphogenesis (Sap9p, Pga24p, Ecm33p) (Fig 2G).

Taken together, our results demonstrate that inhibition of α-1,6-mannose backbone extension by *MNN10* deletion resulted in abnormal cell wall polysaccharides biosynthesis and organization, but had no effect on the anchorage of CWPs including virulence factors.

### Inhibition of α-1,6-mannose backbone extension has no effect on the invasive capacity of *C*. *albicans*

**Fig 3:**
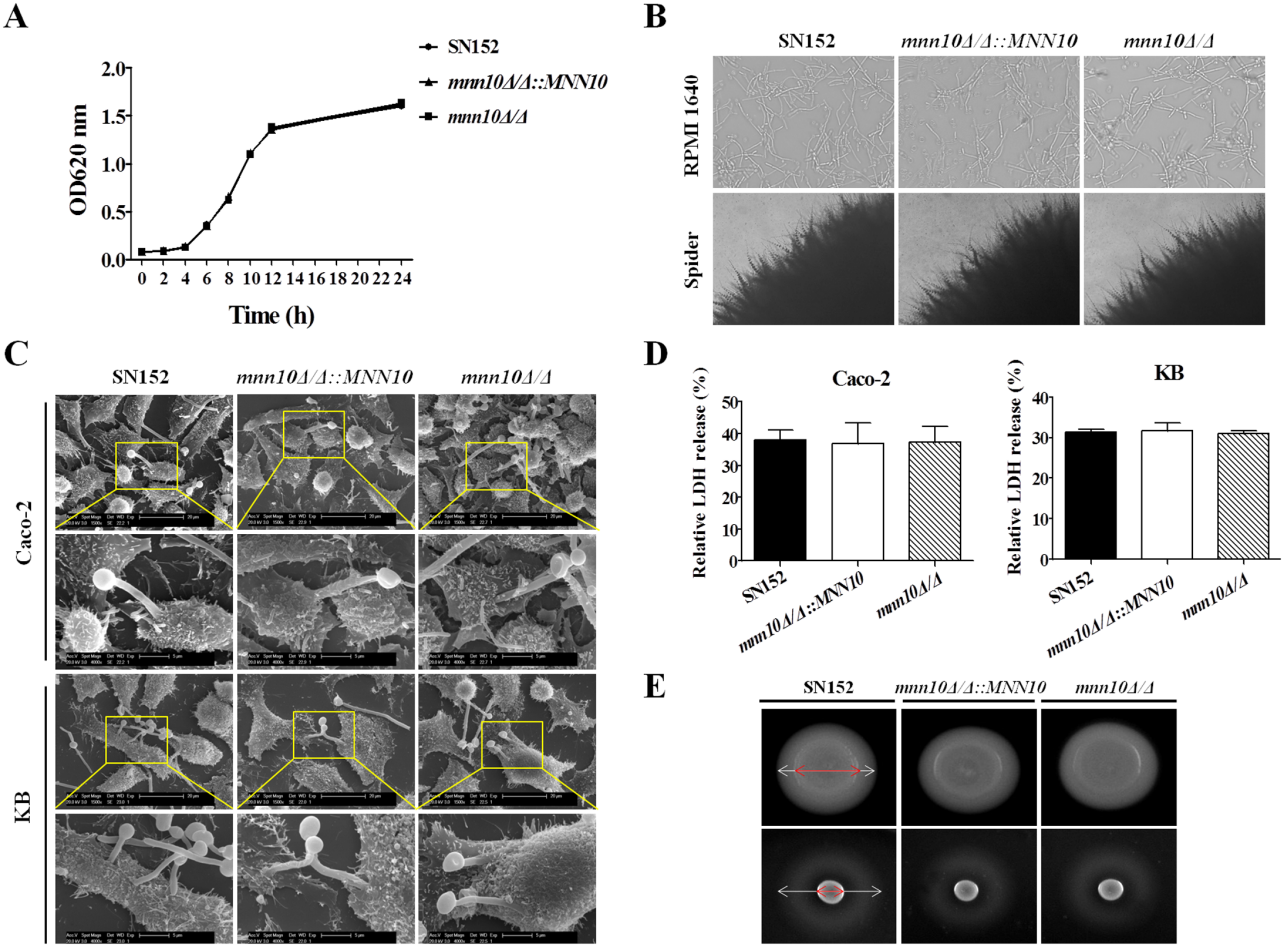
Inhibition of α-1,6-mannose backbone extension in *C*. *albicans* does not affect its invasive capacity *in vitro*. (A) Growth curves of SN152, *mnn10Δ/Δ*::*MNN10* and *mnn10Δ/Δ* strains. (B) Representative photomicrographs of indicated *C*. *albicans* growing in liquid RPMI 1640 culture for 3 h at 37°C or on spider agar media for 5 days at 37°C to induce hyphal form. (C) Representative micrographs of scanning electron microscope of Caco-2 and KB cells invaded or penetrated by SN152, *mnn10Δ/Δ*::*MNN10* and *mnn10Δ/Δ* after 2 h co-incubation (MOI = 1). (D) Epithelial cells damage was determined by assaying LDH release. Relative LDH release from Caco-2 or KB cells was measured after 12 h incubation with *C*. *albicans* (MOI = 0.1). Data are represented as means ± SD from triplicates of one representative experiment of three. *P* > 0.05 (One-way ANOVA with Bonferroni post-test). (E) Extracellular phospholipase and hemolytic activity assays. The phospholipase activity was examined by spotted *C*. *albicans* on egg yolk agar at 37°C for 3 days and observed the width of the zone of precipitation around each colony (top panel). The hemolytic activity was assessed by growing the indicated *C*. *albicans* strains on sugar-enriched sheep blood agar at 37°C for 3 days to observe the presence of a distinct translucent halo around the colonies (lower panel). Representative images are shown from one of three independent experiments.

The composition and organization of the cell wall in *C*. *albicans* plays an important role in the initiation and maintenance of invasive infections. In the context of *MNN10* deletion, we investigated the virulence of *mnn10* mutant strain *in vitro*. Multiplication and yeast-to-hypha transition are the prerequisites of *C*. *albicans* invasive disease [39]. The growth curves obtained demonstrated that *MNN10* did not impact the growth of *C*. *albicans in vitro* (Fig 3A). Moreover, *mnn10* mutant also did not show defective filamentation in either liquid (RPMI1640, 10% serum liquid medium) or solid medium (Spider and Lee’s agar medium) favoring hyphal growth (Figs 3B and S5A).

Adhesion to the epithelium, such as oral and intestinal epithelial cells, and hyphal penetration are the first steps of *C*. *albicans* invasion [40]. Our results indicate that deletion of *MNN10* did not affect the ability of *C*. *albicans* to adhere Caco-2 intestinal epithelial and KB buccal epithelial cells (*P* > 0.05, S5B Fig). Following adhesion, we used scanning electron microscopy (SEM) to evaluate the ability of hyphal penetration and invasion of *mnn10* mutant strain. After a 2 h co-culture, we observed hyphal forms, of all the indicated strains, penetrated Caco-2 and KB cells at the apical face and microvillis attached to the hyphae at the point of penetration, indicating that *MNN10* gene deletion had no effect on the invasion ability of *C*. *albicans in vitro* (Fig 3C). Moreover, no significant difference, in epithelial cell damage, was observed among strains (*P* > 0.05, Fig 3D).

Phospholipases and hemolysins secreted by *C*. *albicans* can also induce host cells damage [41, 42]. We screened extracellular phospholipase and hemolytic activity by growing *C*. *albicans* strains on either egg yolk agar or sheep blood agar. As compared to the parental and revertant strains, *mnn10* mutant induced similar zones of precipitation or clearance around the colonies, indicating that *MNN10* deletion was not significantly associated with phospholipases and hemolysins secretion (Fig 3E).

As such, our results suggest that *MNN10* deletion did not affect the invasive capacity of *C*. *albicans in vitro*.

### *MNN10* gene is required for normal pathogenicity of *C*. *albicans* in a murine systemic candidiasis model

**Fig 4:**
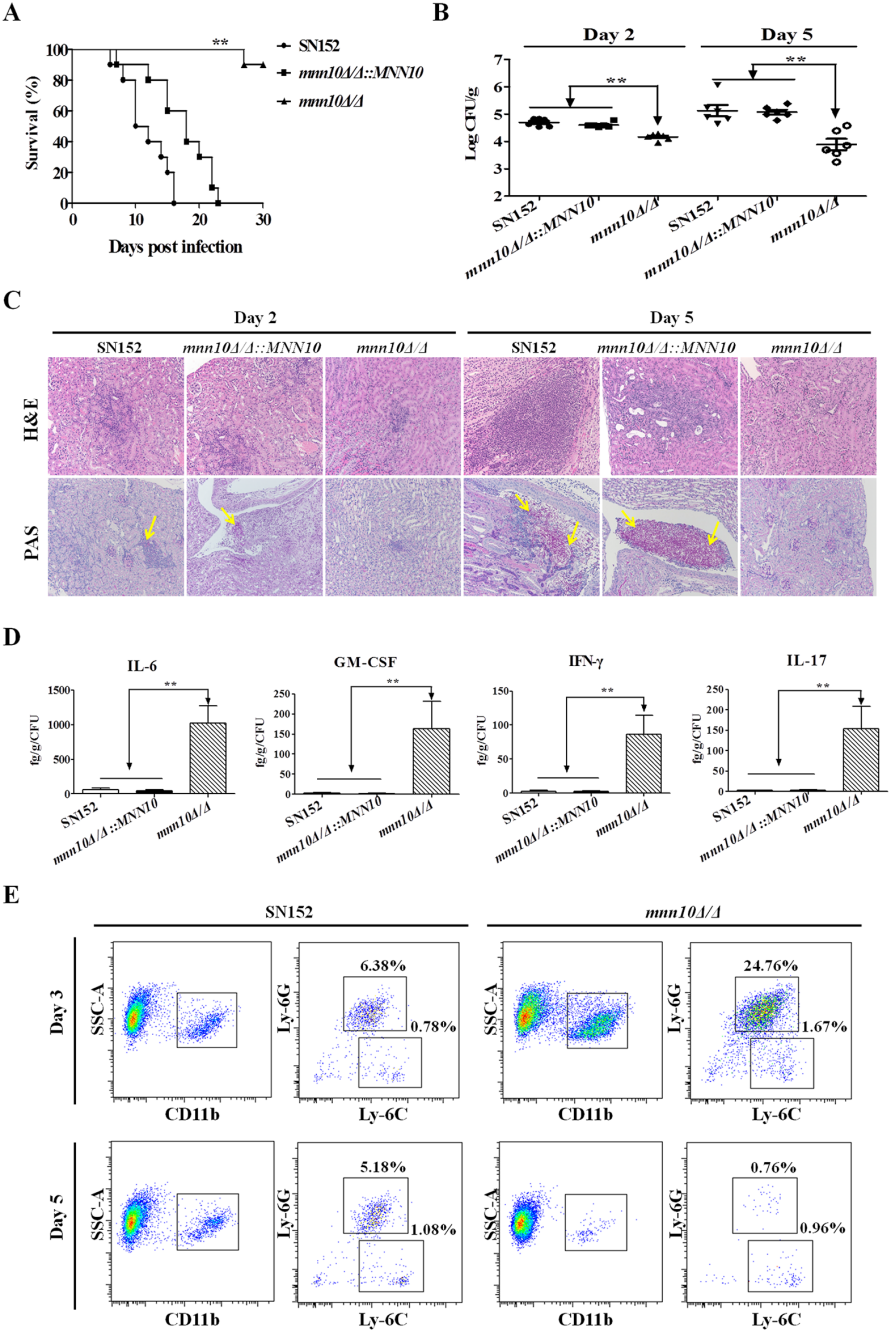
Mnn10 is required for *C*. *albicans* systemic infection. C57BL/6 mice were infected with 5×10^5^ CFU SN152, *mnn10Δ/Δ*::*MNN10* or *mnn10Δ/Δ* strain in 200 μl sterile saline via lateral tail vein. (A) Survival of C57BL/6 mice infected with the indicated strains was monitored for over 30 days (n = 10 per group). Data are representative of three independent experiments. (B) Quantification of the fungal burden in kidney tissues of C57BL/6 mice (n = 6 per group) infected with indicated *C*. *albicans* strains at day 2 and day 5. Data are representative of three independent experiments. (C) Representative H&E (for the inflammatory cells influx and the extent of tissue necrosis) and PAS (for *C*. *albicans*) staining of kidneys from C57BL/6 mice infected with indicated strains at day 2 and 5. Arrows indicate *C*. *albicans* filaments in the tissues. Magnification = 200 ×. (D) ELISA assays for IL-6, GM-CSF, IFN-γ and IL-17 in homogenized kidneys from C57BL/6 mice infected with indicated *C*. *albicans* at day 5 (n = 8 per group). The cytokine levels were normalized to burden of infection in each individually kidney as fg/g tissue/CFU. Data are means ± SD and are representative of three independent experiments. (E) The cellular inflammation in the kidneys of SN152 or *mnn10Δ/Δ* infected mice. SSC^high^CD11b^+^Ly-6C^+^Ly-6G^+^ neutrophils and SSC^high^CD11b^+^Ly-6C^+^Ly-6G^-^ monocytes in the kidneys were detected at Day 3 and Day 5 by flow cytometry. Data are representative images of five mice. *, *P* < 0.05; **, *P* < 0.01 [Log-rank test (A) and Kruskal-Wallis nonparametric One-way ANOVA with Dunns post-test (B, D)].

To examine the effects of α-1,6-mannose backbone of *C*. *albicans* on host infection *in vivo*, we compared the pathogenicity of SN152, *mnn10Δ/Δ*::*MNN10* and *mnn10Δ/Δ* in a murine systemic candidiasis model. Mice infected with *mnn10* mutant strain had a much higher survival rate than those infected with the parental or revertant strain at a lethal dose (5×10^5^ CFU). Over a 30-day observation period, only one mouse infected with the *mnn10* mutant strain died. By contrast, all of the mice infected with the parental or revertant strain died within 30 days (*P* < 0.01, Fig 4A). The median survival analysis demonstrated that the 50% survival limit was attained at 10 days for mice infected with the parental strain and 18 days for the revertant strain, respectively. At day 2 or day 5 post-infection, mice infected with *mnn10* mutant had significantly lower fungal burdens in the kidneys and livers as compared to those infected with either the parental or revertant strain (*P* < 0.01 and *P* < 0.05, respectively; Figs 4B and S6A). Moreover, Hematoxylin and eosin (H&E) staining revealed that during prolonged infections, inflammatory influx and tissue necrosis of the kidneys were aggravated in mice infected with either parental or *MNN10* revertant strains (Fig 4C, top panel). Periodic acid-Schiff (PAS) staining also identified more hyphae in the kidneys of parental or revertant strain infected mice as compared to the *mnn10* mutant strain (Fig 4C, bottom panel). After normalized to organ CFU burden of infection, IL-6, GM-CSF, IFN-γ and IL-17 in the kidneys of *mnn10* mutant infected mice were markedly higher than mice infected with parental or revertant strains (*P* < 0.01, Fig 4D) (the actual cytokine values were shown in S7 Fig).

Furthermore, we performed a flow cytometry analysis to detect cellular inflammation in the kidneys of infected mice. Time-course analysis revealed that *mnn10Δ/Δ* infected mice recruited more SSC^high^CD11b^+^Ly-6C^+^Ly-6G^+^ neutrophils and SSC^high^CD11b^+^Ly-6C^+^Ly-6G^-^ monocytes in the kidney than mice infected with the SN152 strain at day 2 and day 3, respectively (Figs 4E and S8). The cellular inflammation in the kidneys of *mnn10Δ/Δ* infected mice reached a peak at day 3 post-infection, when compared to SN152 infected mice. With decreased fungal burden, neutrophils and monocytes were reduced in the kidney of *mnn10Δ/Δ* infected mice at day 5 post-infection as compared with SN152 infected mice (Fig 4E).

Taken together, the results suggest that inhibition of α-1,6-mannose extension by *MNN10* deletion significantly impacted the pathogenesis of *C*. *albicans* by enhancing the host antifungal defense *in vivo*.

### *MNN10* deletion does not affect the pathogenicity of *C*. *albicans* in athymic nude mice

**Fig 5:**
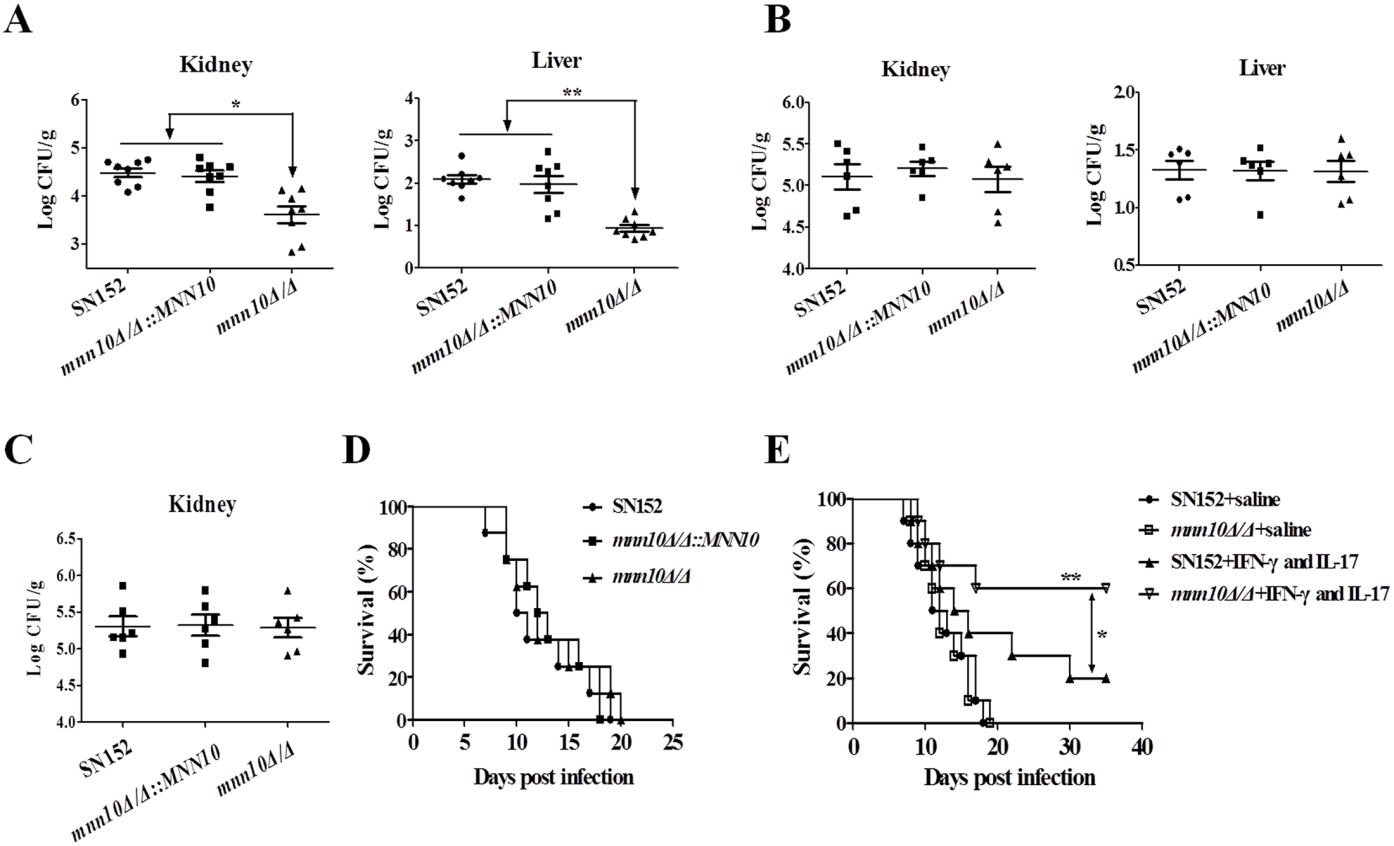
Mnn10 is not required for *C*. *albicans* systemic infection in athymic nude mice. BALB/c mice or athymic nude mice (BALB/c background) were infected with 3×10^5^ CFU of SN152, *mnn10Δ/Δ*::*MNN10* or *mnn10Δ/Δ* strain via lateral tail vein, respectively. The kidney and liver fungal burdens of BALB/c mice (n = 8 per group) (A) and athymic nude mice (n = 6 per group) (B) 5 days after infection. *, *P* < 0.05; **, *P* < 0.01 (Kruskal-Wallis nonparametric One-way ANOVA with Dunns post-test). (C) The kidney fungal burdens of athymic nude mice (n = 6 per group) 10 days after infection. (D) Survival of athymic nude mice infected with the indicated *C*. *albicans* (n = 8 per group). (E) Survival of SN152 and *mnn10Δ/Δ* infected athymic nude mice treated with combination of IFN-γ (100 ng) and IL-17 (100 ng) or the same volume of sterile saline. Data shown are representative of three independent experiments. **, *P* < 0.01 (*mnn10Δ/Δ+*IFN-γ and IL-17 versus *mnn10Δ/Δ*+saline); *, *P* < 0.05 (*mnn10Δ/Δ+*IFN-γ and IL-17 versus SN152+IFN-γ and IL-17) [Kruskal-Wallis nonparametric One-way ANOVA with Dunns post-test (A, B, C) and Log-rank test (D, E)].

To further explore whether host antifungal defense was crucial for the clearance of *mnn10* mutant *in vivo*, we investigated the pathogenicity of *mnn10* mutant strain in BALB/c mice and athymic nude mice (BALB/c background). BALB/c mice infected with *mnn10* mutant displayed significantly lower fungal burdens in the kidneys and livers as compared to those infected with either the parental or revertant strain (*P* < 0.05 and *P* < 0.01, respectively; Fig 5A). However, the results of kidneys and livers fungal burdens indicated *mnn10* was not required for the *C*. *albicans* pathogenesis in athymic nude mice (Fig 5B). To expand upon these findings, longer time course and survival experiments were performed. At day 10 post-infection, no significant difference in the levels of fungal burden of kidneys was observed between mice infected with *mnn10* mutant strain versus those infected with the parental or revertant strains (Fig 5C). Furthermore, there was no significant survival difference among these strains infected mice (Fig 5D).

To confirm the role of elevated IFN-γ and IL-17 in the infected mice (Fig 4D) in clearing *mnn10* mutant strain, we performed an adoptive immunotherapy experiment. The combination of IFN-γ and IL-17 treatment significantly improved the survival of athymic nude mice infected with *mnn10Δ/Δ* (*P* < 0.01, Fig 5E). Moreover, *mnn10* mutant infected mice treated with cytokines had a markedly higher survival rate than SN152 infected mice with the same treatment modality (*P* < 0.05, Fig 5E). Furthermore, we used neutralizing antibodies to IL-17 and/or IFN-γ to elucidate the importance of these cytokines in C57BL/6 mice infected with *mnn10Δ/Δ*. Compared with mice treated with isotype antibody rat IgG1, mice receiving anti-IFN-γ, anti-IL-17A, or both antibodies exhibited significantly higher fungal burdens in the kidneys and livers (S9 Fig) (*P* < 0.05). These results suggest that IFN-γ and IL-17 played an important role in the enhanced host defense against *mnn10* mutant strain *in vivo*.

### Inhibition of α-1,6-mannose backbone extension in *C*. *albicans* induces enhanced host innate immune response

**Fig 6:**
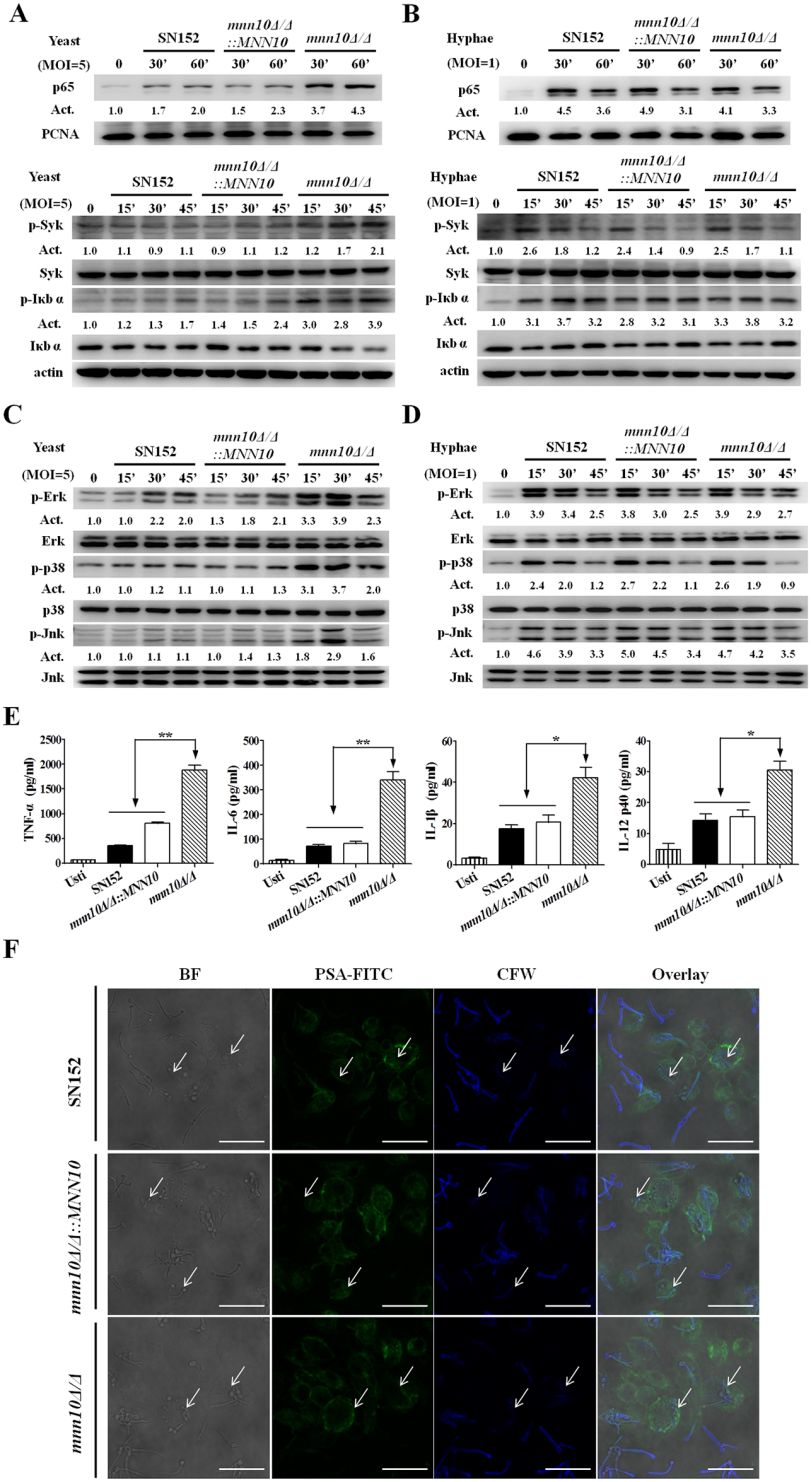
α-1,6-mannose backbone inhibition in *C*. *albicans* yeasts, but not hyphae, could induce an increased immune responses. (A and B) Thioglycollate-elicited peritoneal macrophages were stimulated with UV-inactivated SN152, *mnn10Δ/Δ*::*MNN10* and *mnn10Δ/Δ* yeasts (MOI = 5) (A) or hyphae (MOI = 1) (B) for the indicated times. The nuclear extracts (top panel) and total cell lysates (lower panel) were subjected to immunoblotting analysis with the indicated antibodies of NF-κB signaling. (C and D) Thioglycollate-elicited peritoneal macrophages were challenged with UV-inactivated SN152, *mnn10Δ/Δ*::*MNN10* and *mnn10Δ/Δ* yeasts (MOI = 5) (C) or hyphae (MOI = 1) (D) for the indicated times. The total cell lysates were subjected to immunoblotting with the indicated antibodies of MAPK signaling. Numbers between blots indicate activity (Act) of phosphorylation of MAPK or NF-κB pathways, as measured by densitometry. (E) ELISA results for cytokines TNF-α, IL-6, IL-1β and IL-12p40 in cell supernatants of thioglycollate-elicited peritoneal macrophages, which were stimulated with the indicated *C*. *albicans* yeasts (MOI = 5) for 6 h. Usti, unstimulated. Data are means ± SD of triplicates from one representative experiment of three. Usti, unstimulated. *, *P* < 0.05; **, *P* < 0.01 (One-way ANOVA with Bonferroni post-test). (F) Phagocytosis of *C*. *albicans* by thioglycollate-elicited peritoneal macrophages. Live *C*. *albicans* was co-cultured with the macrophages grown on coverslips in multiwell plates for 90 min. After staining with CFW (1 μg/ml) and PSA-FITC (20 μg/ml) for 10 min, the samples were viewed by confocal laser scanning microscope directly. Scale bar represents 10 μm. Arrows indicate the internalized *C*. *albicans* cells inaccessible to staining with CFW. Bright field (BF), fluorescein isothiocyanate-conjugated pisum sativum agglutinin (PSA-FITC), calcofluor white (CFW) and overlay are shown individually.

Our results suggested that the enhanced host antifungal immunity was the main factor that contributed to the diminished virulence of *mnn10* mutant strain. Thus, we further investigated the myeloid cell recognition and response to *mnn10* mutant using a macrophages-*C*. *albicans* interaction model. We found that *mnn10* mutant yeast cells induced more nuclear translocation of NF-κB (p65), phosphorylation of Syk and IκBα, together with IκBα degradation in thioglycolate-elicited peritoneal macrophages (Fig 6A). Consistently, *mnn10* mutant yeast cells also induced more ERK phosphorylation, p38 phosphorylation, and JNK phosphorylation in macrophages than the parent parental or revertant strains (Fig 6C). We also detected significantly higher levels of inflammatory cytokines including TNF-α, IL-6, IL-1β and IL-12p40 in macrophages induced by *mnn10Δ/Δ* yeast cells (Fig 6E). However, no differences in NF-κB and MAPK signaling activation were observed in macrophages stimulated by hyphal forms of the parental, *mnn10* mutant and revertant strains, the same as the production of proinflammatory cytokines (Figs 6B, 6D and S10).

**Fig 7:**
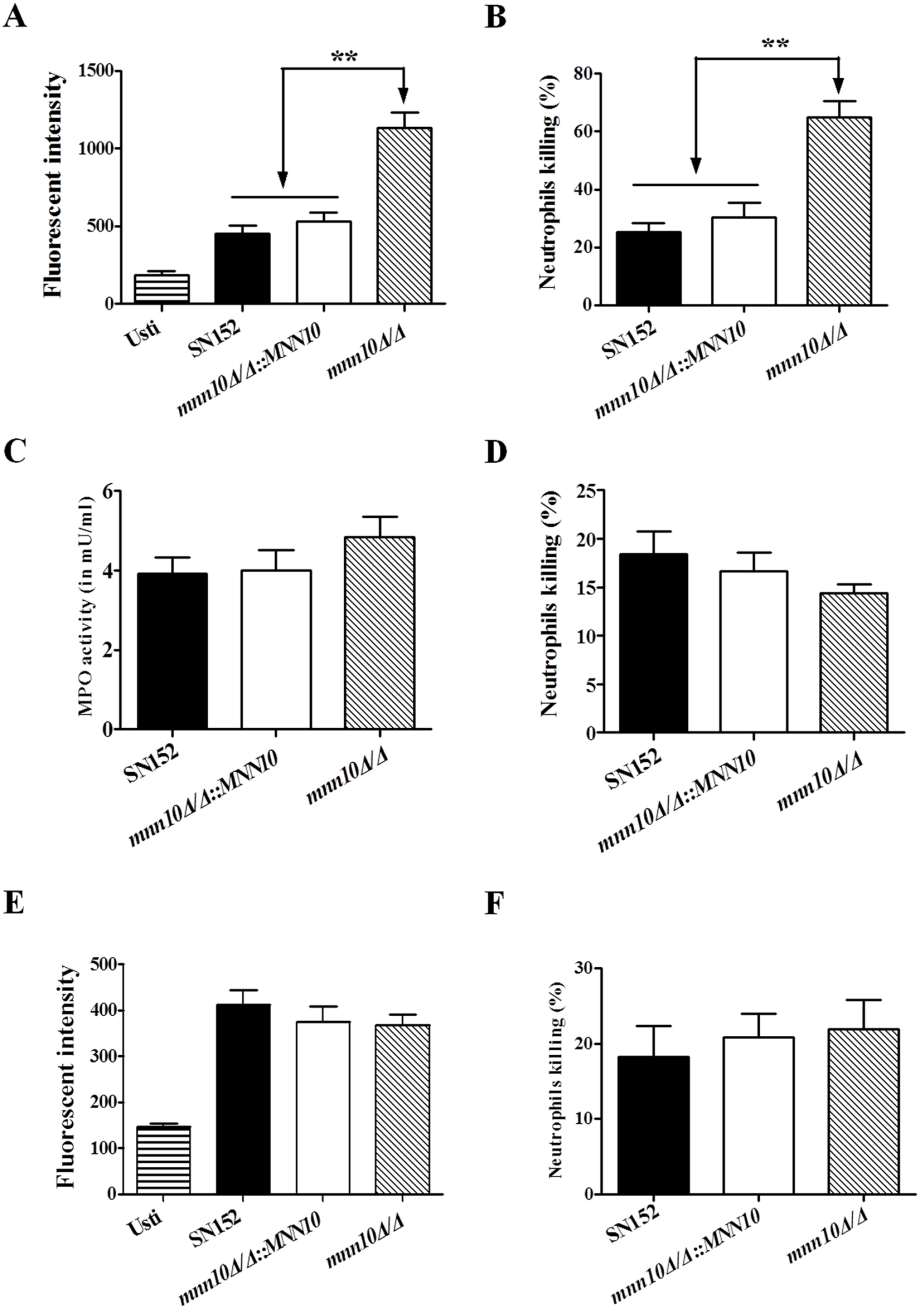
Neutrophils killed *mnn10* mutant more efficiently with an augmented respiratory burst. (A, E) Neutrophils respiratory burst assay. The cellular reactive oxygen species production of thioglycollate-elicited peritoneal neutrophils were measured after incubation with *C*. *albicans* for 1 h (MOI = 1) with (E) or without (A) 2 mM ascorbic acid. (B, D) Neutrophils killing assay. Neutrophils (6×10^5^ cells) were incubated with 3×10^4^ CFU viable *C*. *albicans* for 1 h with (D) or without (B) 2 mM ascorbic acid. Then the suspension was plated on SDA agar for 48 h to count live *C*. *albicans* colonies. (C) Neutrophils were incubated with the indicated *C*. *albicans* for 1 h (MOI = 1). The cellular level of MPO in neutrophils was measured after incubation with *C*. *albicans*. (F) Neutrophils (6×10^5^ cells) were incubated with 3×10^4^ CFU *C*. *albicans* hyphae for 1 h. Then the suspension was plated on SDA agar to count live *C*. *albicans* colonies. Data shown are means ± SD of triplicates from one representative experiment of three. Usti, unstimulated. **, *P* < 0.01 (One-way ANOVA with Bonferroni post-test).

We performed a macrophage phagocytosis assay to investigate whether *mnn10* mutant was differentially taken up from the parental or revertant strain. Our results suggest that no significant difference of the macrophage phagocytosis was visually appreciated between *mnn10* mutant, and the parental or revertant strains in the initial infection stage (Figs 6F and S11). However, we found that thioglycollate-elicited peritoneal neutrophils could produce significantly more ROS and thus destroy *mnn10Δ/Δ* more efficiently (Fig 7A and 7B). While neutrophils can target pathogens in modalities such as myeloperoxidase (MPO), no significant difference in the intracellular MPO activity of neutrophils stimulated by *mnn10* mutant versus parental or revertant strain was observed (Fig 7C). To determine whether the enhanced killing was dependent on ROS production, we scavenged ROS by 2 mM L-ascorbic acid in the co-culture medium, and found that the ability of neutrophils to eliminate *mnn10Δ/Δ* was diminished with decreased ROS (Fig 7D and 7E). However, we found that removal of *mnn10* mutant hyphae by neutrophils was similar in parental or revertant strains (Fig 7F).

**Fig 8:**
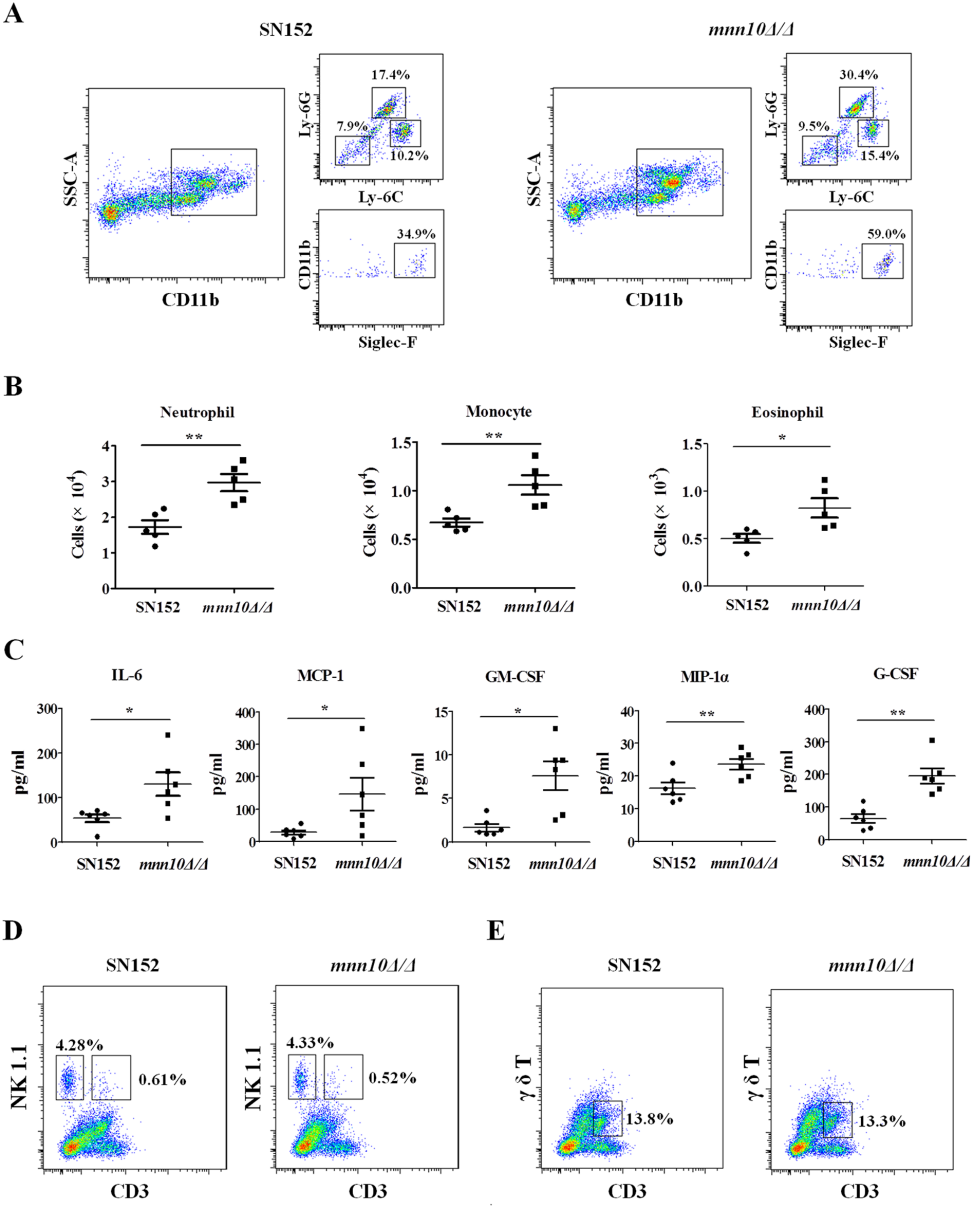
*Mnn10* mutant stimulated enhanced inflammatory responses *in vivo*. (A, B, C) C57BL/6 mice were intraperitoneal infected with 5×10^5^ UV-inactivated *C*. *albicans* of SN152 or *mnn10Δ/Δ* strain for 4 h. (A) Flow cytometry for SSC^high^CD11b^+^Ly-6C^+^Ly-6G^+^ neutrophils, SSC^high^CD11b^+^Ly-6C^+^Ly-6G^-^ monocytes and SSC^high^CD11b^+^Siglec-F^+^ eosinophils in the peritoneum. Data are representative images of five mice. (B) Scatter plots of myeloid cell subsets in the peritoneum cavities (n = 5 per group). (C) ELISA assays for cytokines, chemokines and growth factors in lavage fluid from the inflamed peritoneal cavities (n = 6 per group). Data are representative of three independent experiments. (D, E) C57BL/6 mice were intraperitoneal infected with 5×10^5^ live SN152 or *mnn10Δ/Δ* strain for 2 days. CD3^-^NK1.1^+^ NK cells (D), CD3^+^NK1.1^+^ NKT cells (D), and CD3^+^γ/δ T^+^ cells (E) in the peritoneum were detected by flow cytometry. Data are representative images of five mice. MCP-1, chemokine CCL2; MIP-1α, chemokine CCL3; GM-CSF, granulocyte-monocyte colony-stimulating factor; G-CSF, granulocyte colony-stimulating factor. *, *P* < 0.05; **, *P* < 0.01 [Mann-Whitney nonparametric *t*-test (B, C)].

We further explored how enhanced leukocytes recognition of *mnn10* mutant strain affected inflammation response *in vivo* using a peritoneal infection model. Mice were injected intraperitoneally with *C*. *albicans*, and flow cytometry performed 4 h later revealed that *mnn10Δ/Δ* infected mice recruited more inflammatory cells in the peritoneum than SN152 infected mice, including SSC^high^CD11b^+^Ly-6C^+^Ly-6G^+^ neutrophils, SSC^high^CD11b^+^Ly-6C^+^Ly-6G^-^ monocytes and SSC^high^CD11b^+^Siglec-F^+^ eosinophils (Fig 8A and 8B). The enhanced inflammatory cell recruitment was also associated with increased production of specific cytokines and growth factors such as IL-6, MCP-1, MIP-1α, G-CSF and GM-CSF (Fig 8C). However, this analysis failed to reveal significant difference in the inflammatory cells, including CD3^-^NK1.1^+^ NK cells, CD3^+^NK1.1^+^ NKT cells, and CD3^+^γ/δ T^+^ cells, between *mnn10Δ/Δ* and SN152 infected mice (Fig 8D and 8E).

### Enhanced host antifungal immunity induced by *mnn10* mutant is Dectin-1 dependent

**Fig 9:**
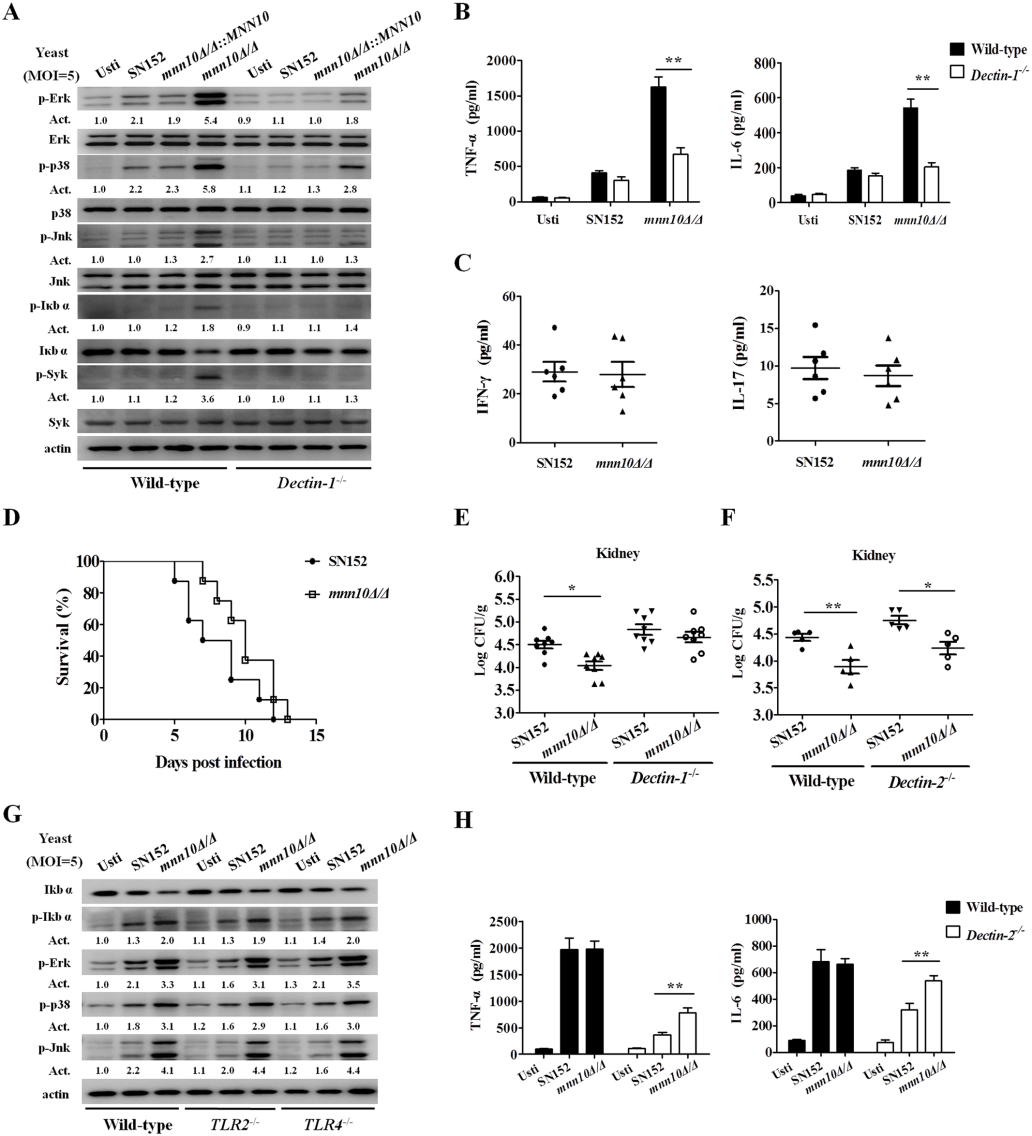
Inflammatory responses, stimulated by *C*. *albicans mnn10* mutant, were Dectin-1 dependent. (A) Thioglycollate-elicited peritoneal macrophages from wild-type or Dectin-1-deficient mice were stimulated with UV-inactivated *C*. *albicans* yeast SN152, *mnn10Δ/Δ*::*MNN10* or *mnn10Δ/Δ* (MOI = 5) for 30 minutes. The total cell lysates were then analyzed by immunoblotting with the indicated antibodies. Numbers between blots indicate activity (Act) of phosphorylation of MAPK or NF-κB pathways, as measured by densitometry. (B) Thioglycollate-elicited peritoneal macrophages from wild-type or Dectin-1-deficient mice were stimulated with the UV-inactivated *C*. *albicans* yeasts (MOI = 5) for 6 h. The amount of TNF-α and IL-6 in supernatants was determined by ELISA. Data are means ± SD of triplicates. (C) ELISA assays for IFN-γ and IL-17 in homogenized kidney from infected Dectin-1-deficient mice at day 5 (n = 6 per group). (D) Survival of Dectin-1-deficient mice infected with 5×10^5^ CFU *C*. *albicans* of SN152 or *mnn10Δ/Δ* strain (n = 8 per group). (E and F) The kidneys fungal burden of Dectin-1-deficient mice (E) or Dectin-2-deficient mice (F) infected with 3×10^5^ *C*. *albicans* at day 5. (G) Wild-type, TLR2-, and TLR4-deficient thioglycollate-elicited peritoneal macrophages were stimulated with UV-inactivated SN152 or *mnn10Δ/Δ* strain of *C*. *albicans* yeast cells (MOI = 5) for 30 min for preparing cell lysates. Samples were subjected to immunoblotting analysis using indicated antibodies. (H) Thioglycollate-elicited peritoneal macrophages from wild-type or Dectin-2-deficient mice were stimulated with the UV-inactivated *C*. *albicans* hyphae (MOI = 1) for 6 h. The amount of TNF-α and IL-6 in supernatants was determined using ELISA. Data shown are representative of three independent experiments. Usti, unstimulated. *, *P* < 0.05; **, *P* < 0.01 [Two-way ANOVA with Bonferroni post-test (B, H); Mann-Whitney nonparametric *t*-test (C); Log-rank test (D); Kruskal-Wallis nonparametric One-way ANOVA with Dunns post-test (E, F)].

Several PRRs, such as TLRs and CLRs, are involved in host defense during *C*. *albicans* infection. We hypothesized that the enhanced host immune response induced by *mnn10* mutant may be attributed to the cell wall β-(1,3)-glucan exposure. We stimulated thioglycollate-elicited peritoneal macrophages from wild-type or Dectin-1-deficient mice with *mnn10* mutant yeasts, and found that activation of NF-κB and MAPK signaling was defective in Dectin-1-deficient macrophages (Fig 9A). Consequently, the *mnn10* mutant yeasts could not significantly increase the production of inflammatory cytokines such as TNF-α and IL-6 in Dectin-1-deficient macrophage cells (Fig 9B). The removal of *mnn10* mutant by neutrophils from Dectin-1-deficient mice was similar in parental or revertant strains (S12A Fig). Moreover, no significant difference in the inflammatory cytokines, including IL-6, GM-CSF, IFN-γ and IL-17, were detected between the kidneys of *mnn10Δ/Δ* and SN152 infected Dectin-1-deficient mice (Figs 9C and S12B). The survival curves indicated that *mnn10* mutant strain presented similar pathogenicity with the parental strain SN152 in Dectin-1-deficient mice (Fig 9D). Dectin-1-deficient mice had similar kidney or liver fungal burdens when infected with the parental SN152 or *mnn10Δ/Δ* strain (*P* > 0.05, Figs 9E and S6B).

However, our results demonstrated that other PRRs involved in antifungal immunity did not contribute to the enhanced immune responses elicited by *mnn10* mutant. By example, *mnn10* mutant strain presented similar pathogenicity in Dectin-2 deficient mice when compared to SN152 in the wild type mice (*P* < 0.05, Figs 9F and S6C). Dectin-2 deficiency had no effect on the inflammatory cytokines production such as TNF-α and IL-6 in macrophages, and TLR2 or TLR4 deficiency had no effect on the activation of NF-κB and MAPK signaling in macrophages when challenged with *mnn10* mutant (Fig 9G and 9H).

Taken together, these data suggest that the enhanced immune response induced by α-1,6-mannose backbone inhibition in *C*. *albicans* was Dectin-1 dependent.

## Discussion

During *C*. *albicans* infection, both yeast cells and hyphae can be found in infected organs or tissues, and innate immune cells discriminate them using different PRRs to elicit a protective immune response [43]. Previous studies have shown that the mannan structure of *C*. *albicans* plays an important role in the development of invasive infection. Here we first determined that the cell wall α-1,6-mannose backbone maintained the pathogenicity of *C*. *albicans* by preventing host, Dectin-1 mediated, recognition of β-(1,3)-glucan. These results highlight a previously unappreciated relationship between cell wall mannan structure and pathogenicity of *C*. *albicans*.

Several genes are involved in the biosynthesis of the cell wall mannan in *C*. *albicans*. Cell wall mannan structure mutant strains, induced by deletion of several genes including *OCH1*, *MNN2* and *MNN5*, often represent a less pathogenic strain *in vivo* [9, 11, 12]. Herein, we determined that Mnn10 has α-1,6-mannosyltransferase activity, and is responsible for α-1,6-mannose backbone biosynthesis in *C*. *albicans* (Fig 1). We also highlighted the role of *MNN10* in pathogenicity of *C*. *albicans*. Our studies using *mnn10* null mutant strain demonstrated that *MNN10* is required for *C*. *albicans* pathogenicity during a systemic candidiasis model in mice (Fig 4).

Infection is mediated by the interplay between a pathogen’s ability to invade host, versus the host attempts to recognize and destroy the pathogen. Several cell wall proteins on the surface of *C*. *albicans* act as virulence factors to invade host [39, 44]. Cell wall proteomic analysis indicated that *MNN10* deletion had no effect on virulence factors of *C*. *albicans* (Fig 2G). Several steps, including adhesion to the epithelium, epithelium penetration and invasion by hyphae, vascular dissemination, and endothelial colonization, are involved in the development of invasive candidiasis [2]. However, the data from our study *in vitro* indicated that the *mnn10* mutant was not defective in its invasive capacity (Fig 3). Therefore, we hypothesize enhanced immune recognition of the mutant strain by the host, rather than decreased virulence, contributed to the attenuated pathogenicity. The normal pathogenicity of *mnn10* mutant in athymic nude mice (BALB/c background) further confirmed our hypothesis (Fig 5B, 5C and 5D). The difference of *mnn10* mutant clearance in BALB/c mice versus athymic nude mice may be attributed to thymus, which is an important organ for the differentiation and maturation of T lymphocytes. Both Th1 and Th17 cells mediate host protection against *C*. *albicans* infection [19, 45]. IFN-γ and IL-17 are the key cytokines produced by Th1 and Th17 cells, which recruit neutrophils and macrophages to destroy the pathogen. IFN-γ and IL-17 elevation, and corresponding neutrophil responses were observed in the kidneys of *mnn10* mutant infected mice, indicating that the *mnn10* mutant could stimulate stronger antifungal response (Fig 4D and 4E). Although the source of IFN-γ and IL-17 can be from innate lymphocytes, our results suggest that elevated levels of IFN-γ and IL-17 elicited by *mnn10* mutant *in vivo* are likely not derived from innate lymphocytes such as NK cells, NKT cells and γ/δ T cells (Fig 8D and 8E). Intracellular cytokine staining analysis revealed that *mnn10Δ/Δ* infected mice induced more IFN-γ-producing and IL-17A-producing α/β T cells than SN152 infected mice (S13 Fig). Therefore, our study suggested that inhibition of α-1,6-mannose backbone extension in *C*. *albicans* induced enhanced T lymphocyte mediated immune response *in vivo*.

The host innate immune cells involved in invading pathogens recognition are predominantly monocytes and neutrophils in circulation and macrophages in infected tissues. Inflammatory cytokines and chemokines can recruit innate immune cells to infected tissues. In a peritoneal infection model, we demonstrated that *mnn10* mutant strain could recruit more neutrophils and monocytes by inducing cytokines and chemokines including IL-6, MCP-1, GM-CSF, MIP-1α and G-CSF in the peritoneal cavity (Fig 8A, 8B and 8C). IL-6 and G-CSF can promote neutrophil production and activation against *C*. *albicans* infection [46]. GM-CSF and MIP-1α are involved in potentiating neutrophil functions and maturation [47, 48]. MCP-1 is a crucial mediator to recruit monocytes in inflammation *in vivo* [49]. Neutrophils contribute to the initial step to kill fungi, and are especially important in neutropenic and immunosuppressed individuals [50]. Our data also determined that neutrophils mainly eliminated *mnn10* mutant strain in a ROS-dependent manner (Fig 7). These results suggest that inhibition of *C*. *albicans* α-1,6-mannose backbone extension by *MNN10* deletion could enhance host innate immune recognition.

Immune recognition could render antigen presenting cells competent to prime T cells, and thereby drive the adaptive Th1 and Th17 immune response. After encountering pathogens, the host macrophages secrete several cytokines, leading to the induction of Th cell differentiation [3]. Our study suggests that *C*. *albicans mnn10* did not play an important role in the initial phagocytosis stage of macrophages (Figs 6F and S11). By contrast, *mnn10* mutant yeast of *S*. *cerevisiae* was poorly taken up by primary macrophages, as compared to the parental strain [51]. We hypothesize that the differential macrophage phagocytosis of *mnn10* mutant might be attributed to the pathogenicity and immunogenicity differences between *C*. *albicans* and *S*. *cerevisiae*. However, we demonstrated that *mnn10* mutant strain elicited enhanced recognition by macrophages. The increased cellular responses of macrophages were associated with NF-κB and MAPK pathway activation, and inflammatory cytokine productions including TNF-α, IL-6, IL-1β and IL-12p40 (Fig 6A, 6C and 6E). TNF-α was involved in the innate immune response against *Candida* infection through promotion of neutrophil production and activation [52]. IL-6 and IL-23 (consisting of IL-12p40 and p19) contributed to Th17 differentiation induced by *C*. *albicans* and *Staphylococcus aureus*, and IL-1β was essential pro-inflammatory regulators of Th17 cells both at priming and effect phase [53]. Therefore, we suggest that the enhanced recognition by innate immune cells could promote Th cell response and thus contributes to host clearance of *mnn10* mutant strain *in vivo*.

The special PAMPs on the surface of *C*. *albicans* could be recognized by PRRs of innate immune cells to initiate the host immunity. The skeletal component of *C*. *albicans* cell wall is based on a core structure of β-(1,3)-glucan that is covalently linked to β-(1,6)-glucan, chitin, and an outer layer of mannoproteins [3]. Recognition of β-(1,3)-glucan by Dectin-1 has been reported to be important in host antifungal defense [22]. However, β-(1,3)-glucan on the surface of *C*. *albicans* was normally shielded by the outer mannan layer from being recognized by Dectin-1 on innate immune cells [2, 27]. Deletion of certain *C*. *albicans* genes, such as the phospholipids phosphatidylserine synthase gene *CHO1* and the histidine kinase gene *CHK1*, could unmask β-glucans of *C*. *albicans*, specifically recognized by Dectin-1 and leading to more host immune responses [30, 31]. Antifungal compounds, such as caspofungin and gepinacin, can also cause the exposure of β-(1,3)-glucan in *C*. *albicans* and elicit a stronger host immune response [29, 54]. Our results demonstrate that inhibition of α-1,6-mannose backbone extension by *MNN10* deletion could unmask the concealed β-(1,3)-glucan in either yeast or hyphal form (Figs 2B, 2E and S4B). The exposure of β-(1,3)-glucan may be due to the fact that the outer structure of cell surface do not adequately conceal the inner layer. The enhanced inflammatory responses stimulated by *mnn10* mutant, including inflammatory signaling activation and cytokine secretion, were markedly down-regulated in Dectin-1-deficient macrophages, suggesting that they were Dectin-1 dependent (Fig 9A and 9B). Moreover, our study indicates that *mnn10* mutant restored its pathogenicity in Dectin-1-deficient mice, further confirming our hypothesis (Fig 9D and 9E). While *mnn10* mutant strain also stimulated enhanced response of macrophage cells from TLR-2, TLR-4 deficient mice or showed less pathogenicity in Dectin-2 deficient mice, indicating the enhanced inflammatory responses induced by *mnn10* mutant strain had little associations with other PRRs involved in host antifungal defense (Fig 9F, 9G and 9H). We concluded that enhanced Dectin-1 dependent immune recognition of *mnn10* mutant strain could induce increased inflammatory cytokines secretion in antigen presenting cells such as macrophages, which further regulated Th1 and Th17 cell differentiation. IFN-γ secreted from Th1 lymphocytes and IL-17 secreted from Th17 lymphocytes ultimately mediated the clearance of *mnn10* mutant strain *in vivo*.

Previous studies have reported that deficiency of Och1 or Mnn2 family involved in mannan biosynthesis of *C*. *albicans* could also lead to β-glucan exposure and decreased mannan [9, 11]. However, Och1 and Mnn2 mutant elicited a host reduced immune response. As we know, PRRs can bind to short oligosaccharides and the precise carbohydrate epitopes to elicit antifungal immunity. We believe these discrepancies may be attributed to the differential effect of these genes on the mannan structure and the precise exposed carbohydrate epitopes of β-glucan. In addition, these previous studies concluded that decreased mannan, rather than β-glucan exposure, was the major PAMP recognized by host immune system in Och1 and Mnn2 mutant [9, 11]. The present study not only indicates that deletion of *MNN10* in *C*. *albicans* results in decreased mannose content and a more remarkable exposure of β-(1,3)-glucan on the cell surface, but also suggests that β-(1,3)-glucan of *mnn10* mutant strain is the major PAMP and induced enhanced Dectin-1 dependent immune response.

In conclusion, we first identified that Mnn10 as an α-1,6-mannosyltransferase, which is involved in the cell wall α-1,6-mannose backbone extension and maintained pathogenicity of *C*. *albicans* by evading host Dectin-1 mediated antifungal immunity. In addition, there are no mammalian homologs of Mnn10 protein, thus our results provide a new potential antifungal therapeutic strategy for modulating the host immune response to *C*. *albicans*.

## Materials and Methods

### Ethics statement

All mouse experimental procedures were performed in accordance with the Regulations for the Administration of Affairs Concerning Experimental Animals approved by the State Council of People’s Republic of China. The protocol was approved by the Institutional Animal Care and Use Committee of Tongji University (Permit Number: TJLAC-014-013).

### Mice

Female C57BL/6 mice, BALB/c nude mice and athymic nude mice (BALB/c background) were obtained from Shanghai Laboratory Animal Center of the Chinese Academy of Sciences. TLR2 deficient and TLR4 deficient mice (C57BL/6 background) were purchased from Shanghai Biomodel Organism Science & Technology Development Company. Dectin-1-deficient (*Clec7a* ^-/-^) mice were kindly provided by Dr. Gordon D. Brown (the mice were backcrossed for nine generations on the C57BL/6 background) and Dectin-2-deficient (*Clec4n* ^-/-^) mice were kindly provided by Dr. Yoichiro Iwakura (C57BL/6 background) [55, 56].

### Cell lines, reagents and antibodies

KB buccal epithelial carcinoma cell line and Caco-2 intestinal epithelial cell line were purchased from BIOK&KM. Alcian Blue, ANTS, HF-pyridine, percoll, calcofluor white (CFW), fluorescein isothiocyanate-conjugated pisum sativum agglutinin (PSA-FITC) and dihydrorhodamine 123 were purchased from Sigma-Aldrich. Antibodies against phospho-ERK, p38, phospho-p38, JNK, phospho-JNK, phospho-IκBα, Syk, phospho-Syk, p65, and PCNA were purchased from Cell Signaling Technologies. Antibodies against ERK, IκBα and β-actin were from Santa Cruz Biotechnology. Alexa-488-labeled and Cy3-labeled goat anti-mouse antibodies were purchased from Life Technologies. Antibody against β-(1,3)-glucan was purchased from Biosupplies Inc. The following antibodies, along with the appropriate isotype controls were used in flow cytometry: peridinin-chlorophyll-protein-complex anti-Ly-6C (clone HK1.4, Biolegend), Alexa Fluor 647 Siglec-F (clone E50-2440, BD Pharmingen), phycoerythrin-Cy7-conjugated anti-CD11b (clone M1/70, Biolegend), phycoerythrin-conjugated anti-Ly-6G (clone 1A8, Biolegend), fluorescein isothiocyanate-conjugated anti-CD3ε (clone 145-2C11, Biolegend), allophycocyanin anti-NK-1.1 (clone PK136, Biolegend) and allophycocyanin anti-TCR γ/δ (clone GL3, Biolegend).

### Strain growth conditions and construction

All strains were maintained on SDA agar plates (1% peptone, 4% dextrose, and 1.8% agar) and grown in YPD broth (1% yeast extract, 2% peptone, and 2% dextrose) at 30°C. For hyphal growth, *C*. *albicans* yeast cells were cultured in RPMI 1640 medium [10.4 g RPMI 1640 (Gibco BRL), 34.5 g morpholinepropanesulfonic acid (Sigma), and 2.0 g NaHCO_3_, pH 7.0, in 1 liter double-distilled water sterilized by filtration] at 37°C for 3 h.

To construct *MNN10* null mutant strain (*mnn10Δ/Δ*), the entire open reading frame of *MNN10* was deleted from the parental strain SN152 by homologous recombination of auxotrophic markers *HIS1* and *LEU2* using a fusion-PCR-based strategy as previously described [57, 58]. To construct *MNN10* revertant strain (*mnn10Δ/Δ*::*MNN10*), the fusion fragment containing *MNN10* ORF and *C*. *albicans SAT1-*flipper cassette was transformed into *mnn10Δ/Δ* and the *SAT* marker was subsequently looped out as described previously [59]. All of the strains and the primers used in this study were listed in S2 and S3 Tables.

### Expression and purification of Mnn10 protein

The non-transmembrane region of *C*. *albicans MNN10* encoding amino acid residues 70 to 335 was cloned into pMAL-p5X (NEB) including MBP tag. And then the plasmid was transformed into BL21 (DE3) pLysS cells for expressing MBP-fused Mnn10 protein. The transformants were cultured overnight at 37°C and diluted 1:100 in fresh LB culture. When the medium OD_600_ was up to 0.6 at 37°C, IPTG at a final concentration of 0.1 mM was added and the cells were grown overnight at 16°C. MBP-fused Mnn10 protein was purified by amylose resin (NEB) according to the protocols as previously described [60]. The supernatant was passed through a 0.45 μm filter and bound to amylose resin by gravity flow. Unspecific proteins were washed off by applying 10 column volumes (CVs) of column buffer. The protein of interest was then eluted by elute buffer (column buffer added with 10 mM maltose). The eluate was finally dialyzed against buffer (25 mM Tris-HCl, pH7.5) for 2 h at room temperature.

### α-1,6-Mannosyltransferase activity assay *in vitro*

The mannosyltransferase activity assay was performed according to the method as described previously [61, 62]. The purified MBP-fused Mnn10 protein (8 μg) was subjected to the buffer (10 mM MnCl_2_, 50 mM HEPES, 10 mM α-1,6-D-mannobiose and 5 mM GDP-D-Mannose, pH 7.2). The α-1,6-mannose extended reaction was incubated at 30°C for 1 h. After labeled with 0.75 μmol 8-Aminonaphthalene-1,3,6-trisulfonic acid (ANTS), the products were separated by electrophoresis on 40% polyacrylamide gels and detected by UV light on a transilluminator (3500 R, Tanon). The reaction products were then digested with α-1,6-mannosidase (NEB) overnight at 37°C according to manufacturer’s instructions, and analyzed by FACE.

### Alcian Blue binding assay

The Alcian Blue binding assay was carried out as described previously [35]. 1.5×10^7^ exponentially growing *C*. *albicans* cells were washed with 0.02 M HCl, and then incubated with 30 μg/ml Alcian Blue for 10 min at room temperature. The supernatant was measured at OD_600_ and the concentration of Alcian Blue was determined by reference to a standard curve. The amount of dye bound to *C*. *albicans* cells were calculated by subtracting the amount of dye in the supernatant.

### Cell surface hydrophobicity

Exponentially growing *C*. *albicans* cells were washed and resuspended in PBS buffer (OD_600_ = 1.0), and 0.75 ml cyclohexane was then added to the above 3 ml cell suspension. The mixtures were vortexed for 3 min and settled for 20 min at room temperature, the OD_600_ of the aqueous phase was measured. The relative hydrophobicity was measured as [(OD_600_ of the control minus OD_600_ after octane overlay)/OD_600_ of the control] × 100% [63].

### Isolation and analysis of cell wall proteins (CWPs)

The covalent and non-covalent CWPs were isolated as previously described [64, 65]. The major types of covalently linked CWPs are glycosylphosphatidylinositol anchored proteins (GPI-APs) and proteins with internal repeats (Pir proteins). GPI-CWPs were released by resuspending the cell wall debris in undiluted HF-pyridine and incubated at 0°C for 3 h. Pir proteins were specifically released by incubating cell wall debris with 30 mM NaOH at 4°C for 16 h. The non-covalent CWPs of *C*. *albicans* were extracted by SDS buffer (50 mM Tris-HCl, 2% SDS, 100 mM EDTA, and 10 mM DTT, pH 8.0). The whole CWPs were then mixed and further digested by trypsin for the analysis of LC-MS/MS on high-resolution instruments (LTQ-Orbitrap XL and Velos, Thermo Fisher). Raw files were processed by MaxQuant soft for peptide/protein identification and quantification.

### Confocal laser scanning microscopy

To stain β-(1,3)-glucan of the cell wall, exponentially growing *C*. *albicans* yeast cells were washed in PBS (for hyphal form assays, 1×10^6^ *C*. *albicans* cells were cultured in RPMI 1640 medium at 37°C for 3 h on a microscope slide in a six-well plate), and then incubated with anti-β-(1,3)-glucan antibody overnight at 4°C and then stained by Cy3-labeled antibody for 1 h at 30°C. To stain mannan and chitin of the cell wall, *C*. *albicans* yeast cells or hyphae were washed in PBS and incubated in the dark with 50 μg/ml ConA to stain for α-mannopyranosyl or 30 μg/ml CFW for chitin for 30 min. The above stained cells were washed and scanned at 63 × magnification with confocal laser scanning microscope (TCS SP5; Leica). Micrograph pictures were then acquired and analyzed by LAS AF Lite program.

### Transmission electron microscopy

5×10^7^ exponentially growing *C*. *albicans* cells were washed in PBS and then fixed in 4 ml fixative solution (3% paraformaldehyde, 3.6% glutaraldehyde, pH 7.2) for 24 h at 4°C. After post-fixation of samples with 1% phosphotungstic acid for 2 h, they were washed by distilled water, block-stained with uranyl acetate, dehydrated in alcohol, immersed in propylenoxide, and embedded in glycide-ether. Ultrathin sections were observed under a transmission electron microscope (Hitachi H-800, Japan) at 120 kV.

### Assessment of virulence *in vitro*

To measure the growth curve of *C*. *albicans*, exponentially growing cells were washed and resuspended in fresh YPD broth (OD_600_ = 0.1), and then the optical density was determined at the indicated time point. To observe the hyphal growth, *C*. *albicans* cells were sub-cultured at 37°C in either RPMI 1640 medium plus 10% (vol/vol) heat-inactivated fetal calf serum (FCS) or spider solid medium.

Phospholipase activity and hemolytic activity of *C*. *albicans* strains were screened as described previously [66, 67]. Briefly, the suspension of yeast cells were spotted on egg yolk agar or sugar-enriched sheep blood agar and incubated at 37°C for 3 days. The phospholipase activity of each strain was observed by measuring the width of zone of precipitation around the colony. The presence of a distinct translucent halo around the colony indicated positive hemolytic activity.

### *C*. *albican*s invasion and epithelial cell damage assays

The Caco-2 or KB cells were grown as approximate 80%-90% confluent monolayer in MEM medium with 20% (vol/vol) heat-inactivated FCS. For SEM, the cells were grown on 8 mm diameter glass coverslips. Each coverslip was infected with 1×10^6^ live *C*. *albicans* yeast cells. After 2 h of infection, the cells were gently washed with PBS prior to 1% OsO_4_ and then examined using a XL-30 scanning electron microscope (Philips, Holland) as described previously [68].

For the cell damage assay, 80%-90% confluent monolayer of Caco-2 or KB cells was infected with 1×10^5^ live *C*. *albicans* yeast cells for 12 h, respectively. Lactate dehydrogenase (LDH) in the medium released from control or infected epithelial cells was determined by LDH Assay kit (Beyotime, China) according to the manufacturer’s instructions. Maximal LDH release was obtained by adding 0.1 ml of 1% Triton X-100 to each well and vigorously disrupting the epithelial layers 1 h before the end of incubation period. The relative LDH activity was measured as [(OD_490_ of infected cells minus OD_490_ of the control)/(OD_490_ of maximal LDH release minus OD_490_ of the control)] × 100%.

### Thioglycollate-elicited peritoneal macrophages and neutrophils preparation

Thioglycollate-elicited peritoneal macrophages and neutrophils were isolated as previously described [69]. Briefly, C57BL/6 mice were injected intraperitoneally with 2 ml 3% (wt/vol) thioglycollate (Merck). Peritoneal cells were collected by washing with PBS containing 0.5 mM EDTA 14 h later and 72 h later, for neutrophils and macrophages isolation, respectively. The cells were cultured in RPMI1640 plus 10% (vol/vol) heat-inactivated FCS.

### Neutrophils killing assay *in vitro*

The killing assay was carried out as described previously [70]. Thioglycollate-elicited peritoneal neutrophils were mixed with live *C*. *albicans* [multiplicity of infection (MOI) = 1:20] in a 24-well plate, and were kept for 1 h at 4°C to settle the cells before being transferred to 37°C for another 1 h. Control plates were kept in parallel at 4°C during the incubation. Then the cells were mixed and plated on SDA agar for counting live *C*. *albicans* colonies for 48 h at 30°C.

For analysis of reactive oxygen species (ROS), the inflammatory cells were co-cultured with *C*. *albicans* (MOI = 1) in RPMI medium containing 10 μM dihydrorhodamine 123 for 1 h at 37°C. After incubation the fluorescent intensity of the oxidized dihydrorhodamine 123 was measured by a multi-mode microplate reader (excitation wavelength, 485 nm; emission wavelength, 538 nm). Cells loaded with dihydrorhodamine 123 but not treated with *C*. *albicans* were used to assess background of ROS production. For analysis of myeloperoxidase (MPO), the neutrophils were lysed by 1% Triton X-100 for 10 min and the MPO activity in neutrophil lysates was measured using an enzyme assay as described previously [71].

### Macrophage-*C*. *albicans* interaction

*C*. *albicans* yeast cells or hyphae were exposed to four doses of 100,000 μjoules/cm^2^ in a CL-1000 Ultraviolet Crosslinker (UVP), with agitation between each dose to treat cells evenly [29]. The thioglycollate-elicited macrophages were stimulated with the UV-inactivated *C*. *albicans* yeasts (MOI = 5) or hyphae (MOI = 1) for the indicated time. Macrophage phagocytosis assay was performed as previously described, exponentially growing *C*. *albicans* cells were washed in PBS buffer and added to the monolayer macrophages (MOI = 5) at the indicated time (30 min, 60 min, 90 min and 120 min). CFW staining was performed for *C*. *albicans* and PSA-FITC staining was performed for macrophages. CFW/PSA-FITC stained samples were scanned immediately at 63 × magnification with confocal laser scanning microscope. Micrograph pictures were acquired and analyzed by LAS AF Lite program.

### Western blotting

The cells were lysed in lysis buffer (250 mM NaCl, 50 mM HEPES, 1 mM EDTA, 1% NP-40, protease inhibitors, pH 7.4) for total cell lysates. For nuclear extracts, cells were lysed in lysis buffer (10 mM KCl, 10 mM HEPES, 0.1 mM EDTA, 0.4% NP-40, protease inhibitors, pH 7.9). The nuclear pellets were harvested, washed with the lysis buffer and resuspended in the extraction buffer (20 mM HEPES, 400 mM NaCl, 1 mM EDTA, protease inhibitors, pH 7.9), and then incubated with vortexing at 4°C for 30 minutes. The cell lysates were subjected to SDS-PAGE, blotted with the indicated primary antibodies and secondary antibodies, and then developed with the chemiluminescence method according to the manufacturer’s instructions (Millipore) using the ECL detection system (GE Healthcare). The densitometry of indicated blot was quantified using Image J software (National Institutes of Health, USA).

### Cytokine production measurement

Concentrations of tumor necrosis factor alpha (TNF-α), interleukin-6 (IL-6), IL-1β and IL-12p40 in cell culture supernatant, gamma interferon (IFN-γ) and IL-17 in tissue homogenates, IL-6, chemokines monocyte chemotactic protein-1 (MCP-1), macrophage inflammatory protein-1α (MIP-1α), and granulocyte and granulocyte-monocyte colony-stimulating factors (G-CSF/GM-CSF) in peritoneal lavage fluid, were measured with commercially available Ready-Set-Go cytokine kits (eBioscience) or cytokine multiplex kits (R&D systems) in triplicate times according to the manufacturer’s instructions.

### Murine systemic candidiasis model

For the *C*. *albicans* infection *in vivo*, groups of C57BL/6 female mice or BALB/c female mice (6–8 weeks) were injected via lateral tail vein with 200 μl of a suspension containing indicated live *C*. *albicans* (5×10^5^ cells for C57BL/6 mice and 3×10^5^ cells for BALB/c mice) in sterile saline. Mice were monitored daily and were killed after 2 days or 5 days of infection. The kidneys and livers were removed, and then homogenized in 0.5 ml PBS for fungal burdens measurement or fixed in 10% neutral formalin for H&E and PAS staining. Supernatants of kidney and livers homogenates were harvested and stored at -80°C for the measurement of cytokine production.

### Cellular inflammation assay in the kidney

C57BL/6 mice were injected with 5×10^5^ CFU *C*. *albicans* and sacrificed at 12 h, 1 day, 2 days, 3 days, 4 days or 5 days post-infection and the kidneys were removed. The kidneys were minced into tissue pieces and digested for 1 h at 37°C. Then the digested tissues were passed through a 70-mm filter, washed and centrifuged in a 40%/70% percoll gradient for leukocytes isolation [72]. The leukocytes at the interphase were then analyzed by flow cytometry (BD FACS).

### Murine peritoneal infection model

C57BL/6 mice were injected intraperitoneally with 5×10^5^ CFU *C*. *albicans* and were killed after 4 h or 2 days. The peritoneal infiltrate was collected by lavage with ice-cold PBS containing 0.5 mM EDTA, and then the red blood cells were lysed. The inflammatory cells were counted and blocked with PBS containing 5% heat-inactivated FCS and 1 mM sodium azide at 4°C. The populations of the cells were analyzed by flow cytometry to determine the leukocyte composition as described before [22].

### Statistical analysis

At least three biological replicates were performed for all experiments unless otherwise indicated. Log-rank test was used to evaluate the equality of survival curves. The two-tailed Student’s *t*-test was used for analysis of two groups and multiple groups were analyzed by one-way analysis of variance with Bonferroni post-tests. For analysis of nonparametrically distributed data, the Mann-Whitney test or Kruskal-Wallis test was used. Statistical significance was set at a p-value in the figures as: *, *P* < 0.05; **, *P* < 0.01.

## Supporting Information

S1 Fig Expression and purification of Mnn10 protein. SDS-PAGE analysis of Mnn10 expressed in *Escherichia coli*. Lane 1, molecular weight markers; lane 2, the culture supernatant of total protein of *E*. *coli* strain; lane 3 and 4, the expressed and purified supernatant of *E*. *coli* strain transformed with an empty vector pMAL-p5X; lane 5 and 6, expression and purification of MBP-fused Mnn10 protein.
(TIF)

S2 Fig Construction of strains. (A) Fusion-PCR-based cassette method for disruption of *MNN10* in two-step homologous recombination and *SAT1* flipper cassette for construction of the reconstituted strain. (B, D) PCR confirmation of *MNN10* gene deletion and its revertant strain by genomic DNA. Genomic DNA from *mnn10* mutant and revertant strains were PCR amplified with the oligonucleotides indicated at the bottom of the figure. (C) Quantitative real-time RT-PCR analysis of *MNN10* in *C*. *albicans* paretal strain SN152 and revertant strain *mnn10Δ/Δ*::*MNN10*. Gene expression is indicated as a fold change relative to SN152. Data are means ± SD of triplicates from one representative experiment of three.
(TIF)

S3 Fig Stress resistance of *C*. *albicans* strains. The parental strain SN152, *mnn10* mutant and revertant strains were spotted in 10-fold dilutions onto YPD agar plates supplemented with the indicated stresses. Plates were incubated for 48 h at 30°C.
(TIF)

S4 Fig Mnn10 was required for cell wall polysaccharides organization in *C*. *albicans* hyphae. Fluorescence micrographs of the cell wall carbohydrate layers from hyphal form of SN152, *mnn10Δ/Δ*::*MNN10* and *mnn10Δ/Δ*, which were stained with ConA-FITC to visualise mannan (A), β-glucan antibody to visualise β-glucan (B). Bright field (BF), fluorescence (FL), and overlay are shown individually. Scale bar represents 10 μm.
(TIF)

S5 Fig Mnn10 was not required for *C*. *albicans* hyphal growth and adherence to host epithelial cells. (A) Exponentially growing *C*. *albicans* cells were incubated in RPMI 1640 medium plus 10% (vol/vol) heat-inactivated fetal calf serum for 3 h, or grew on Lee’s agar media for 5 days at 37°C. Representative photographs were shown. (B) The adherence of *C*. *albicans* to Caco-2 or KB cells was evaluated by co-incubating for 1 h in six-well tissue culture plates, after which the adherent colonies were counted. Data represent mean (± SD) of triplicates from one representative experiment of three.
(TIF)

S6 Fig Liver fungal burdens of mice systemic infected with *C*. *albicans*. (A) Liver fungal burden of wild-type mice infected with 5×10^5^ CFU of the indicated *C*. *albicans* strains at day 2 and day 5. (B) The liver fungal burden of wild-type or Dectin-1 deficient mice infected with 3×10^5^ CFU of the indicated *C*. *albicans* strains at day 5. (C) The liver fungal burden of wild-type or Dectin-2 deficient mice infected with 3×10^5^ CFU of the indicated *C*. *albicans* strains at day 5. Data shown are representative of three independent experiments. **, *P* < 0.01; *, *P* < 0.05 (Kruskal-Wallis nonparametric One-way ANOVA with Dunns post-test).
(TIF)

S7 Fig ELISA assays for IL-6, GM-CSF, IFN-γ and IL-17 in homogenized kidney from *C*. *albicans* infected mice. C57BL/6 mice were infected with 5×10^5^ CFU of SN152, *mnn10Δ/Δ*::*MNN10* or *mnn10Δ/Δ* strain via lateral tail vein at day 2 and day 5 (A top panel, and B) (n = 8 per group). The cytokine levels were normalized to burden of infection in each individually kidney as fg/g tissue/CFU (A, bottom panel). Data are means ± SD and are representative of three independent experiments. *, *P* < 0.05; **, *P* < 0.01 (Kruskal-Wallis nonparametric One-way ANOVA with Dunns post-test).
(TIF)

S8 Fig The cellular inflammation in the kidneys of SN152 or *mnn10Δ/Δ* infected mice. C57BL/6 mice were infected with 5×10^5^ CFU of parental strain SN152 or *mnn10* mutant strain via lateral tail vein. (A) SSC^high^CD11b^+^Ly-6C^+^Ly-6G^+^ neutrophils and SSC^high^CD11b^+^Ly-6C^+^Ly-6G^-^ monocytes in the kidneys were detected at the indicated time by flow cytometry. Data are representative images of five mice. (B) The absolute number of neutrophils and monocytes cells in the kidneys of SN152 or *mnn10* mutant strain infected mice (n = 5 per group). *, *P* < 0.05; **, *P* < 0.01 (Two-way ANOVA with Bonferroni post-test).
(TIF)

S9 Fig The kidney (A) and liver (B) fungal burdens of C57BL/6 mice treated with neutralizing antibodies were determined at day 5 post-infection with *mnn10* mutant strain. Mice (n = 6 per group) were treated with 500 μg of anti-IFN-γ (clone XMG1.2, BioLegend), 100 μg of anti-IL-17A (clone TC11-18H10.1, BioLegend), mixture of anti-IFN-γ and anti-IL-17A, or rat IgG1 per mouse 1 day before and at day 1 and 3 after injection of *mnn10* mutant strain. *, *P* < 0.05 (Kruskal-Wallis nonparametric One-way ANOVA with Dunns post-test).
(TIF)

S10 Fig ELISA results for cytokines TNF-α, IL-6, IL-1β and IL-12p40 in cell supernatants. Thioglycollate-elicited peritoneal macrophages were stimulated with the indicated *C*. *albicans* hyphae (MOI = 1) for 6 h. Usti, unstimulated. Data are means ± SD of triplicates from one representative experiment of three.
(TIF)

S11 Fig Phagocytosis of *C*. *albicans* by macrophages. Live *C*. *albicans* was added to the macrophages grown on coverslips in multiwell plates at the indicated time. After adding CFW (1 μg/ml) and PSA-FITC (20 μg/ml) to the culture medium for 10 min, the samples were viewed by confocal laser scanning microscope directly. Scale bar represents 10 μm. Arrows indicate the internalized *C*. *albicans* cells inaccessible to staining with CFW. Bright field (BF) and fluorescence (FL) are shown individually.
(TIF)

S12 Fig Neutrophil killing and cytokine responses of infected Dectin-1 deficient mice. (A) The thioglycollate-elicited peritoneal neutrophils from Dectin-1 deficient mice (6×10^5^ cells) were incubated with 3×10^4^ CFU *C*. *albicans* for 1 h. Then the suspension was plated on SDA agar to count live *C*. *albicans* colonies. Data are means ± SD of triplicates. (B) ELISA assays for IL-6, GM-CSF in homogenized kidney from infected Dectin-1 deficient mice at day 5 (n = 6 per group). Data are representative of three independent experiments.
(TIF)

S13 Fig The absolute number of IFN-γ-producing cells (A) and IL-17A-producing cells (B) in the spleen of SN152 or *mnn10* mutant strain infected mice. C57BL/6 mice were infected with 5×10^5^ CFU of parental strain SN152 or *mnn10* mutant strain via lateral tail vein (n = 5 per group). Intracellular cytokine IFN-γ and IL-17 from α/β or γ/δ T cells were analyzed after gated on CD3^+^ T cells, and intracellular cytokine signals from NK cells were analyzed after gated on CD3^-^ T cells.
(TIF)

S1 Table CWPs identified by LC-MS/MS in *C*. *albicans*. (DOCX)

S2 Table Strains used in this study. (DOCX)

S3 Table Primers used in this study. (DOCX)
