# Supplementary figures and images for: Mnn10 Maintains Pathogenicity in Candida albicans by Extending α-1,6-Mannose Backbone to Evade Host Dectin-1 Mediated Antifungal Immunity
Source: PLoS Pathog. 2016 May 4;12(5):e1005617. doi: 10.1371/journal.ppat.1005617 (PMC4856274; doi:10.1371/journal.ppat.1005617)

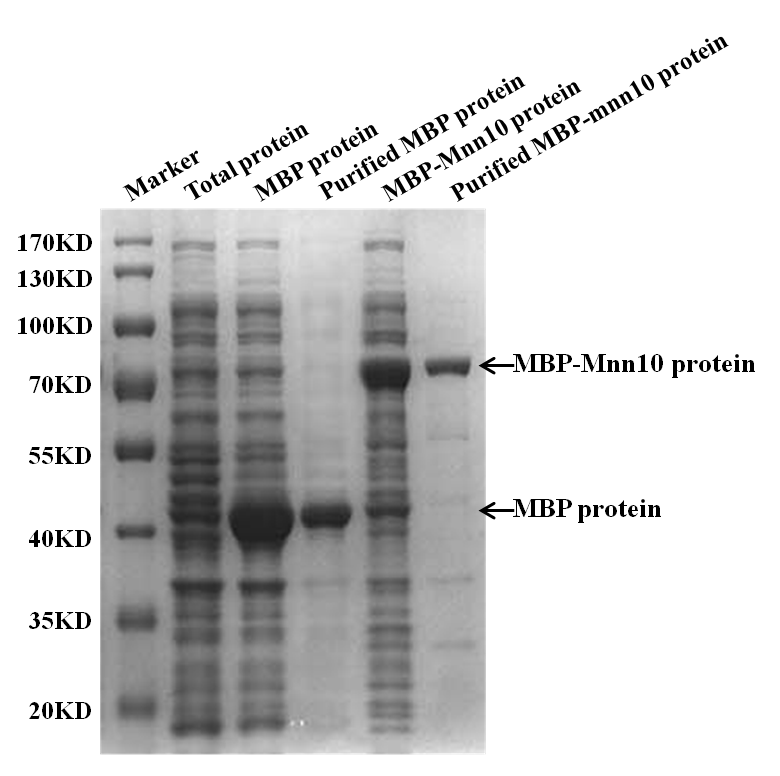

Supplement: S1 Fig — SDS-PAGE analysis of Mnn10 expressed in Escherichia coli. Lane 1, molecular weight markers; lane 2, the culture supernatant of total protein of E. coli strain; lane 3 and 4, the expressed and purified supernatant of E. coli strain transformed with an empty vector pMAL-p5X; lane 5 and 6, expression and purification of MBP-fused Mnn10 protein. (TIF) [file ppat.1005617.s001.tif]

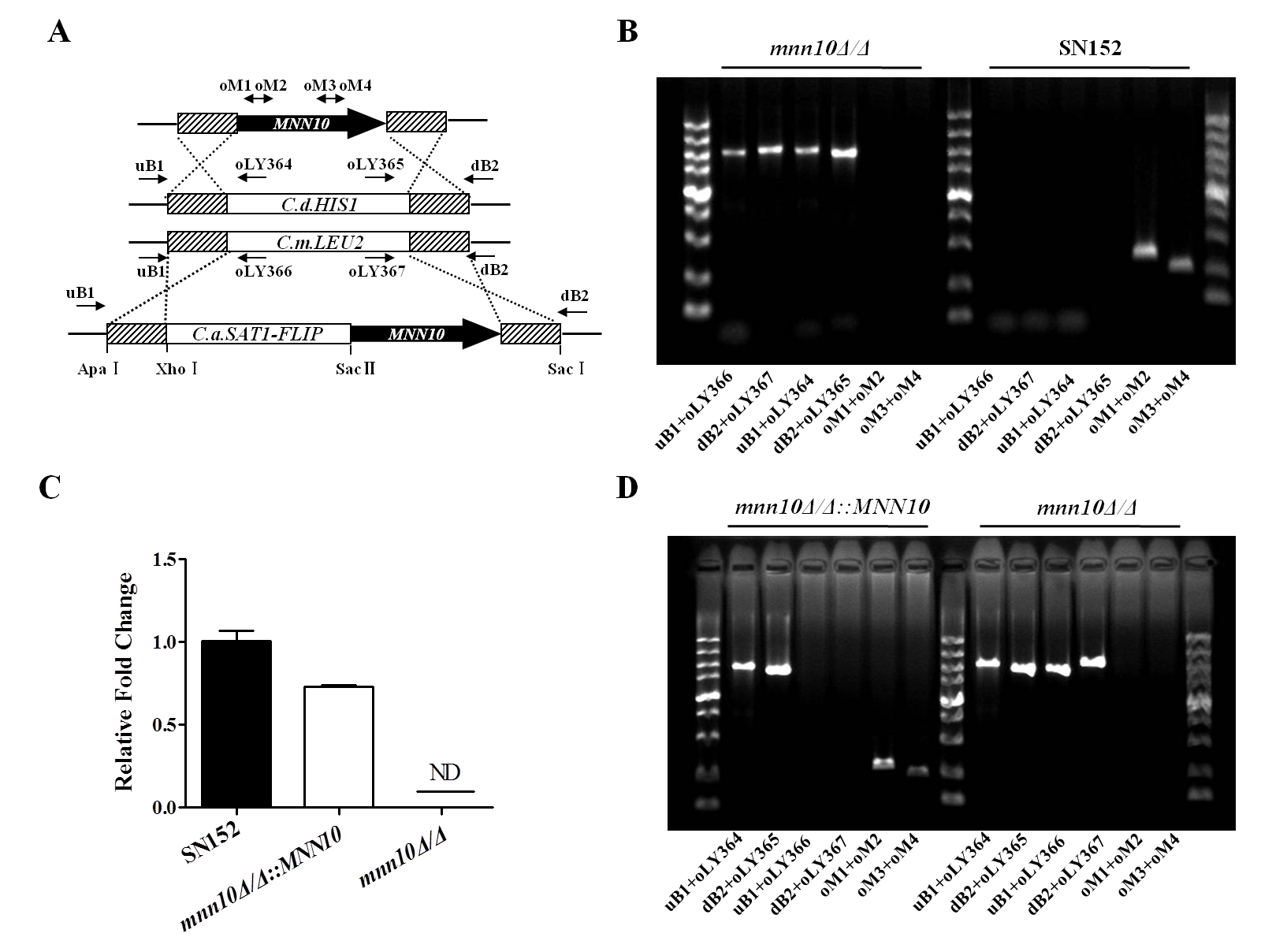

Supplement: S2 Fig — (A) Fusion-PCR-based cassette method for disruption of MNN10 in two-step homologous recombination and SAT1 flipper cassette for construction of the reconstituted strain. (B, D) PCR confirmation of MNN10 gene deletion and its revertant strain by genomic DNA. Genomic DNA from mnn10 mutant and revertant strains were PCR amplified with the oligonucleotides indicated at the bottom of the figure. (C) Quantitative real-time RT-PCR analysis of MNN10 in C. albicans paretal strain SN152 and revertant strain mnn10Δ/Δ::MNN10. Gene expression is indicated as a fold change relative to SN152. Data are means ± SD of triplicates from one representative experiment of three. (TIF) [file ppat.1005617.s002.tif]

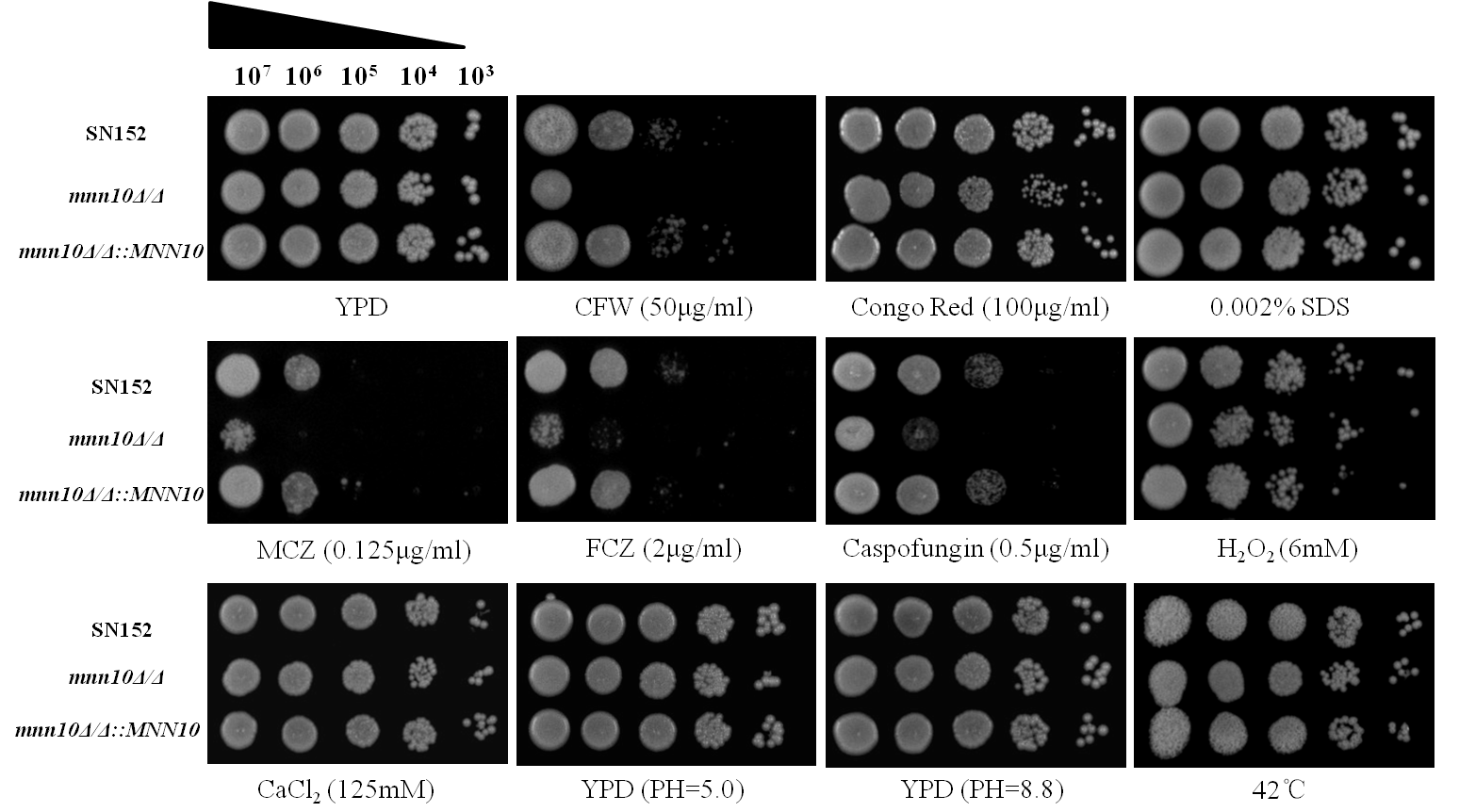

Supplement: S3 Fig — The parental strain SN152, mnn10 mutant and revertant strains were spotted in 10-fold dilutions onto YPD agar plates supplemented with the indicated stresses. Plates were incubated for 48 h at 30°C. (TIF) [file ppat.1005617.s003.tif]

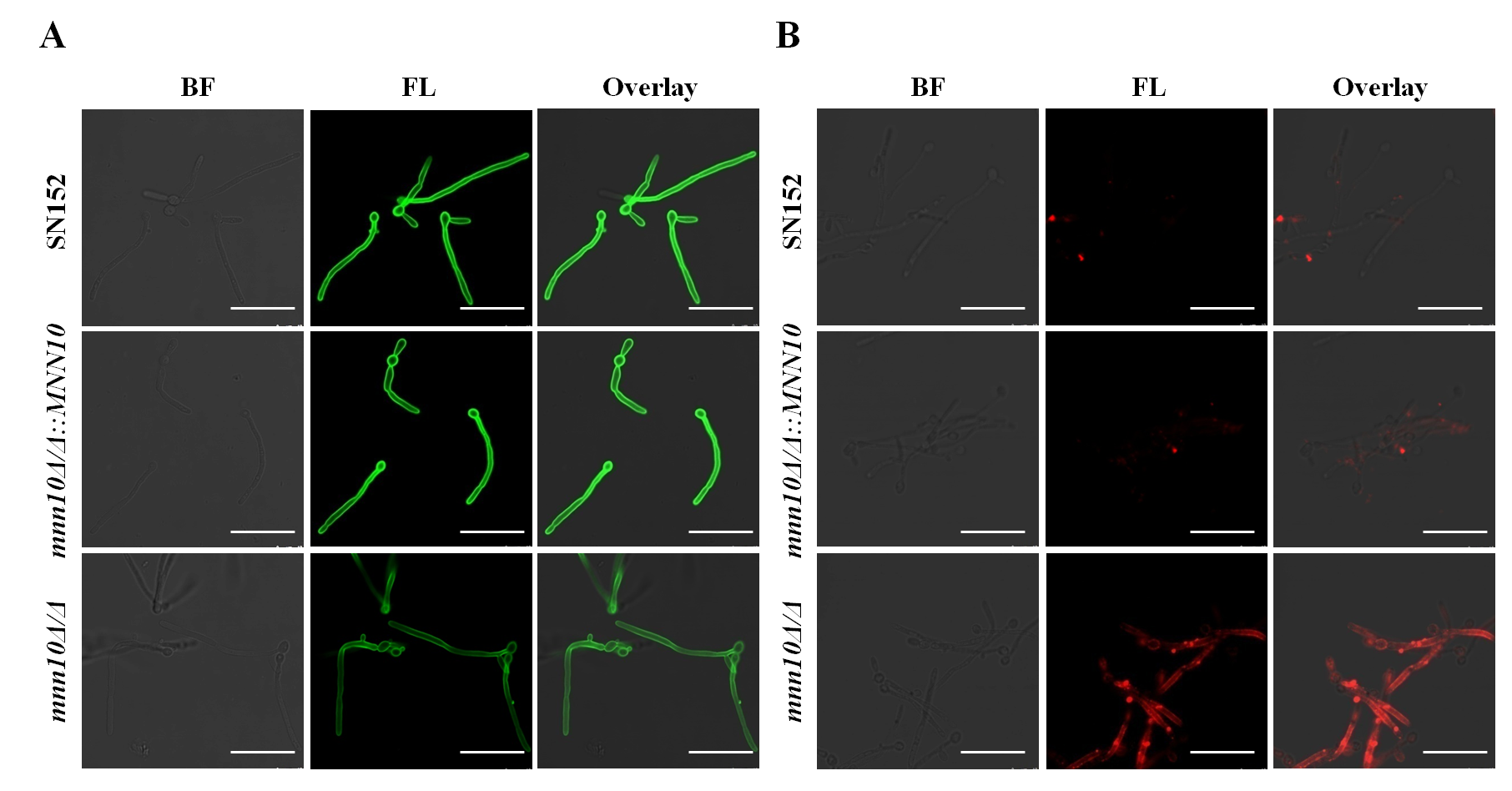

Supplement: S4 Fig — Fluorescence micrographs of the cell wall carbohydrate layers from hyphal form of SN152, mnn10Δ/Δ::MNN10 and mnn10Δ/Δ, which were stained with ConA-FITC to visualise mannan (A), β-glucan antibody to visualise β-glucan (B). Bright field (BF), fluorescence (FL), and overlay are shown individually. Scale bar represents 10 μm. (TIF) [file ppat.1005617.s004.tif]

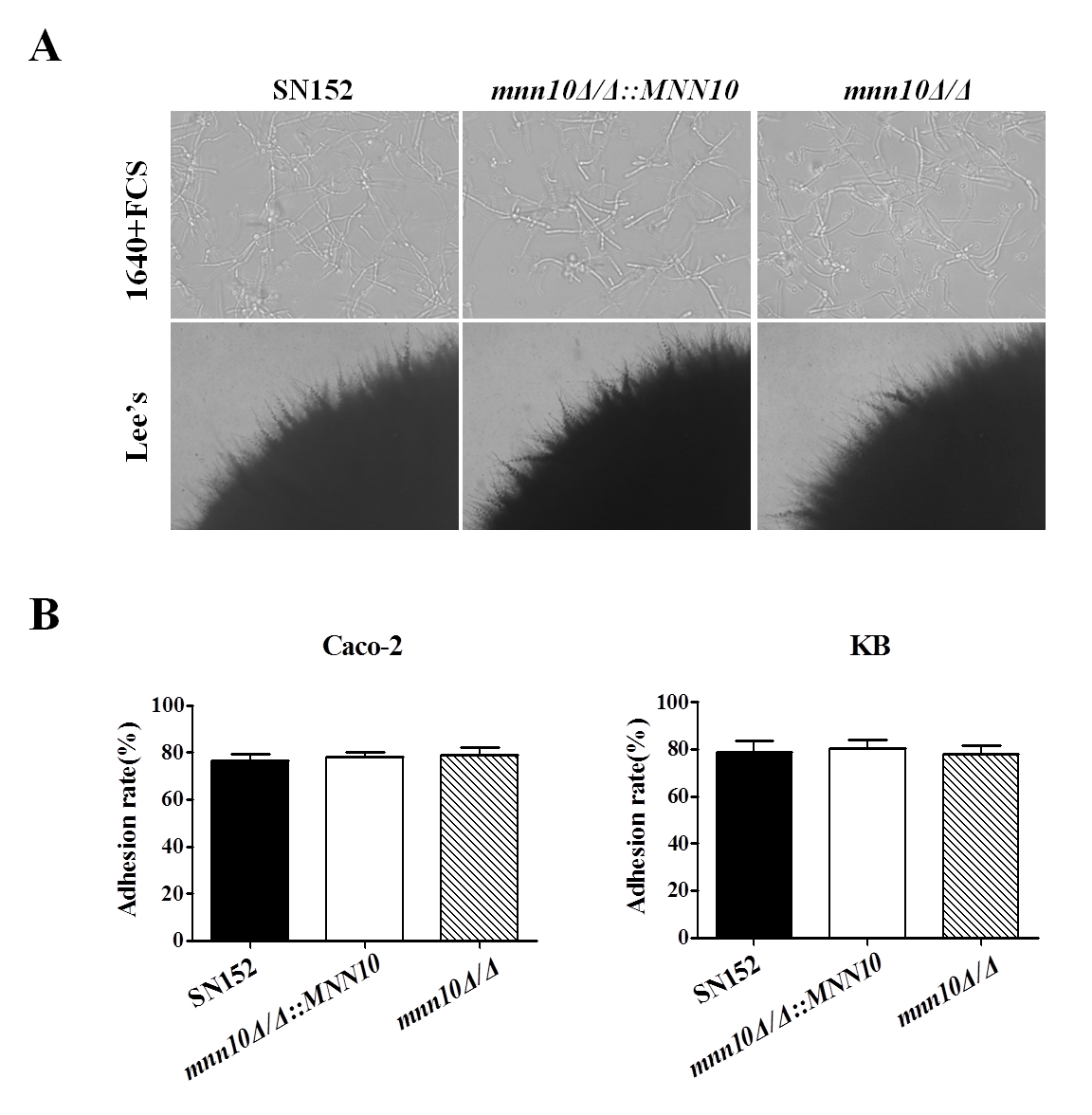

Supplement: S5 Fig — (A) Exponentially growing C. albicans cells were incubated in RPMI 1640 medium plus 10% (vol/vol) heat-inactivated fetal calf serum for 3 h, or grew on Lee’s agar media for 5 days at 37°C. Representative photographs were shown. (B) The adherence of C. albicans to Caco-2 or KB cells was evaluated by co-incubating for 1 h in six-well tissue culture plates, after which the adherent colonies were counted. Data represent mean (± SD) of triplicates from one representative experiment of three. (TIF) [file ppat.1005617.s005.tif]

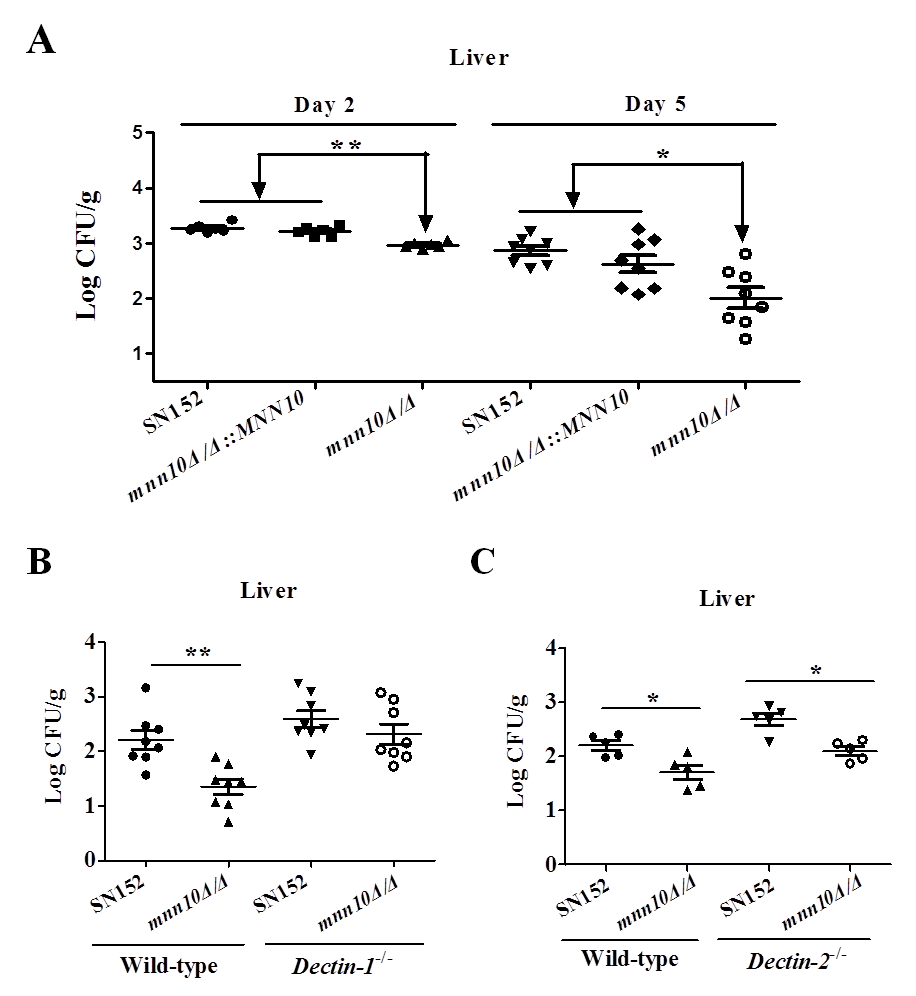

Supplement: S6 Fig — (A) Liver fungal burden of wild-type mice infected with 5×10^5^ CFU of the indicated C. albicans strains at day 2 and day 5. (B) The liver fungal burden of wild-type or Dectin-1 deficient mice infected with 3×10^5^ CFU of the indicated C. albicans strains at day 5. (C) The liver fungal burden of wild-type or Dectin-2 deficient mice infected with 3×10^5^ CFU of the indicated C. albicans strains at day 5. Data shown are representative of three independent experiments. **, P < 0.01; *, P < 0.05 (Kruskal-Wallis nonparametric One-way ANOVA with Dunns post-test). (TIF) [file ppat.1005617.s006.tif]

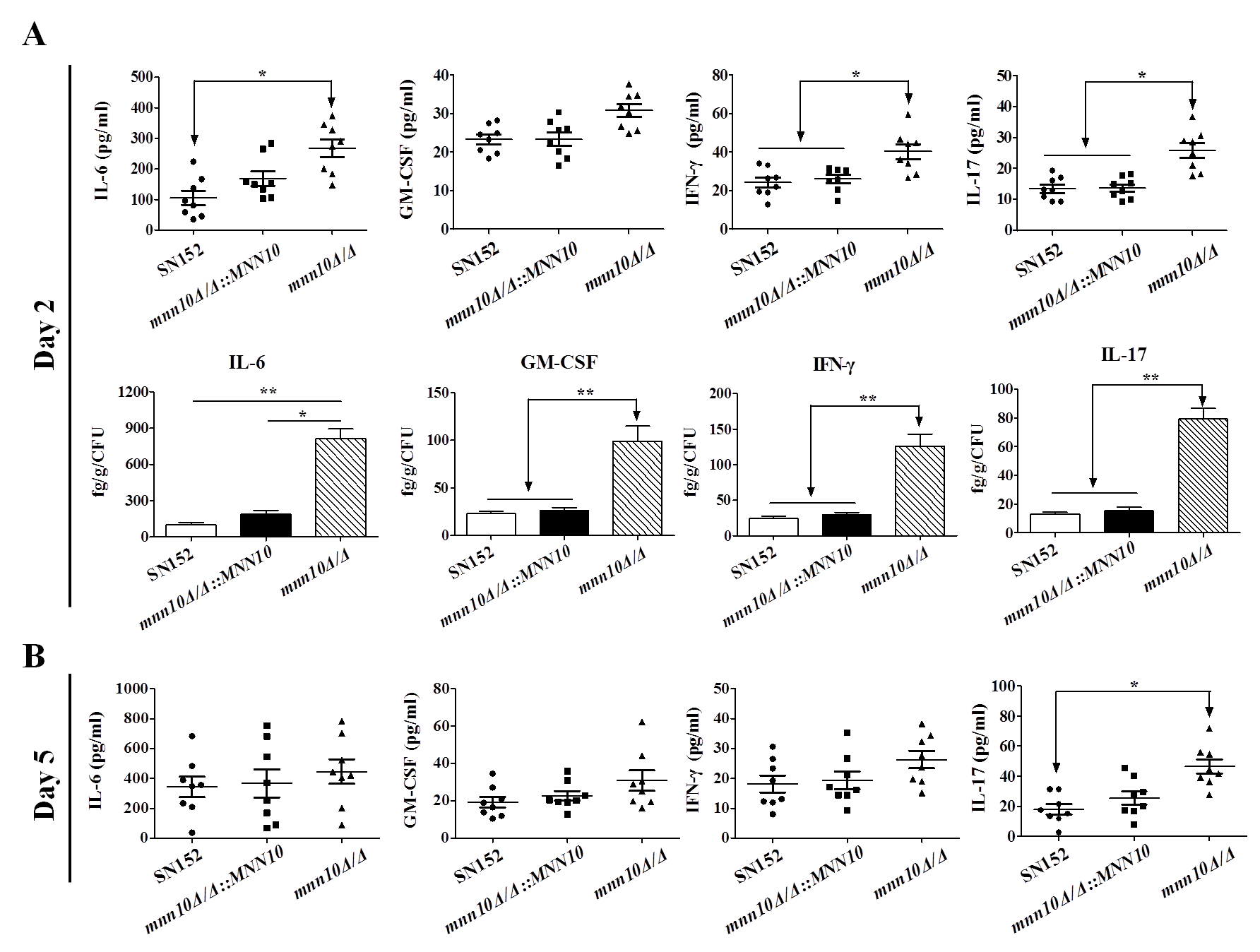

Supplement: S7 Fig — C57BL/6 mice were infected with 5×10^5^ CFU of SN152, mnn10Δ/Δ::MNN10 or mnn10Δ/Δ strain via lateral tail vein at day 2 and day 5 (A top panel, and B) (n = 8 per group). The cytokine levels were normalized to burden of infection in each individually kidney as fg/g tissue/CFU (A, bottom panel). Data are means ± SD and are representative of three independent experiments. *, P < 0.05; **, P < 0.01 (Kruskal-Wallis nonparametric One-way ANOVA with Dunns post-test). (TIF) [file ppat.1005617.s007.tif]

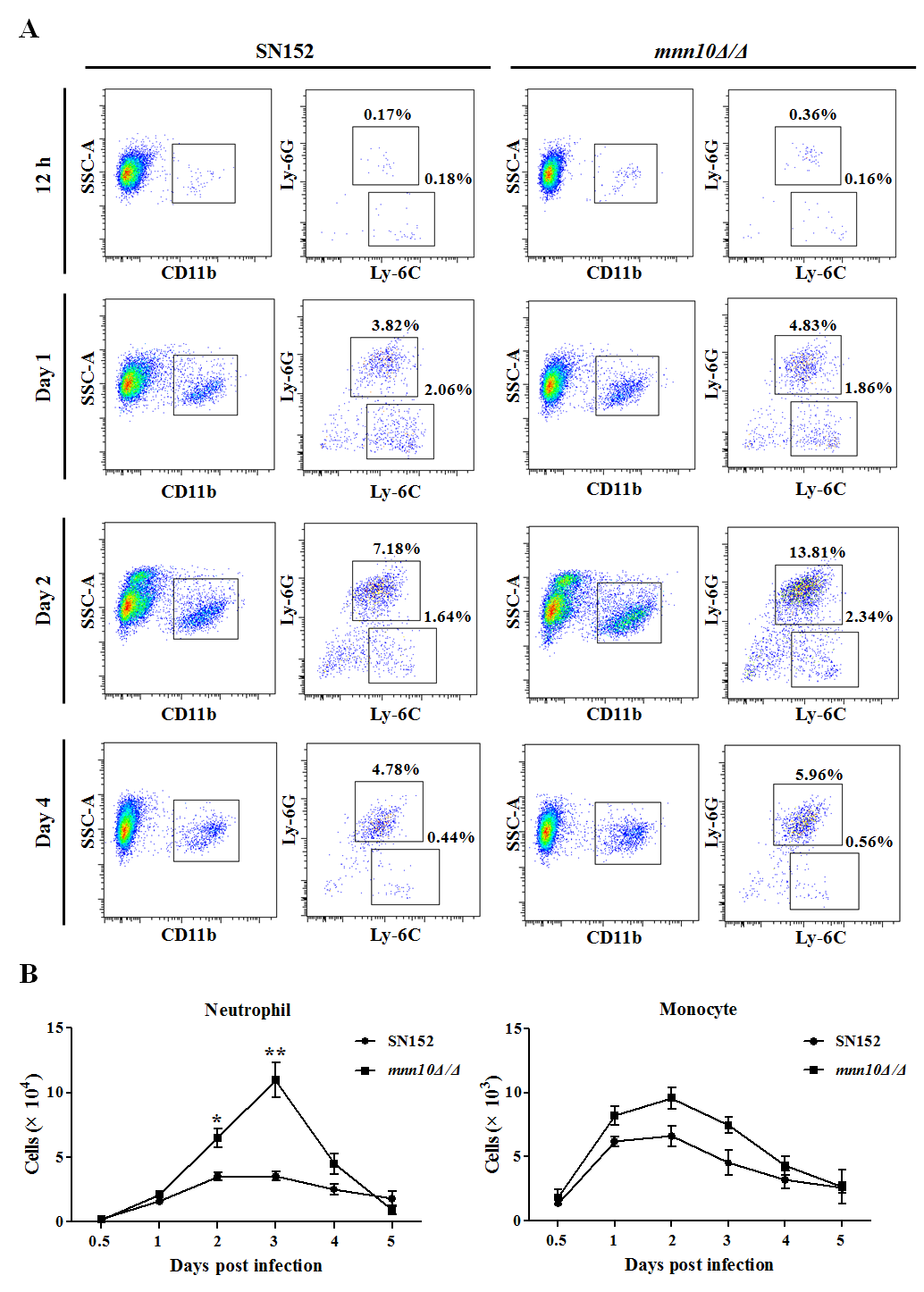

Supplement: S8 Fig — C57BL/6 mice were infected with 5×10^5^ CFU of parental strain SN152 or mnn10 mutant strain via lateral tail vein. (A) SSChighCD11b+Ly-6C+Ly-6G+ neutrophils and SSChighCD11b+Ly-6C+Ly-6G- monocytes in the kidneys were detected at the indicated time by flow cytometry. Data are representative images of five mice. (B) The absolute number of neutrophils and monocytes cells in the kidneys of SN152 or mnn10 mutant strain infected mice (n = 5 per group). *, P < 0.05; **, P < 0.01 (Two-way ANOVA with Bonferroni post-test). (TIF) [file ppat.1005617.s008.tif]

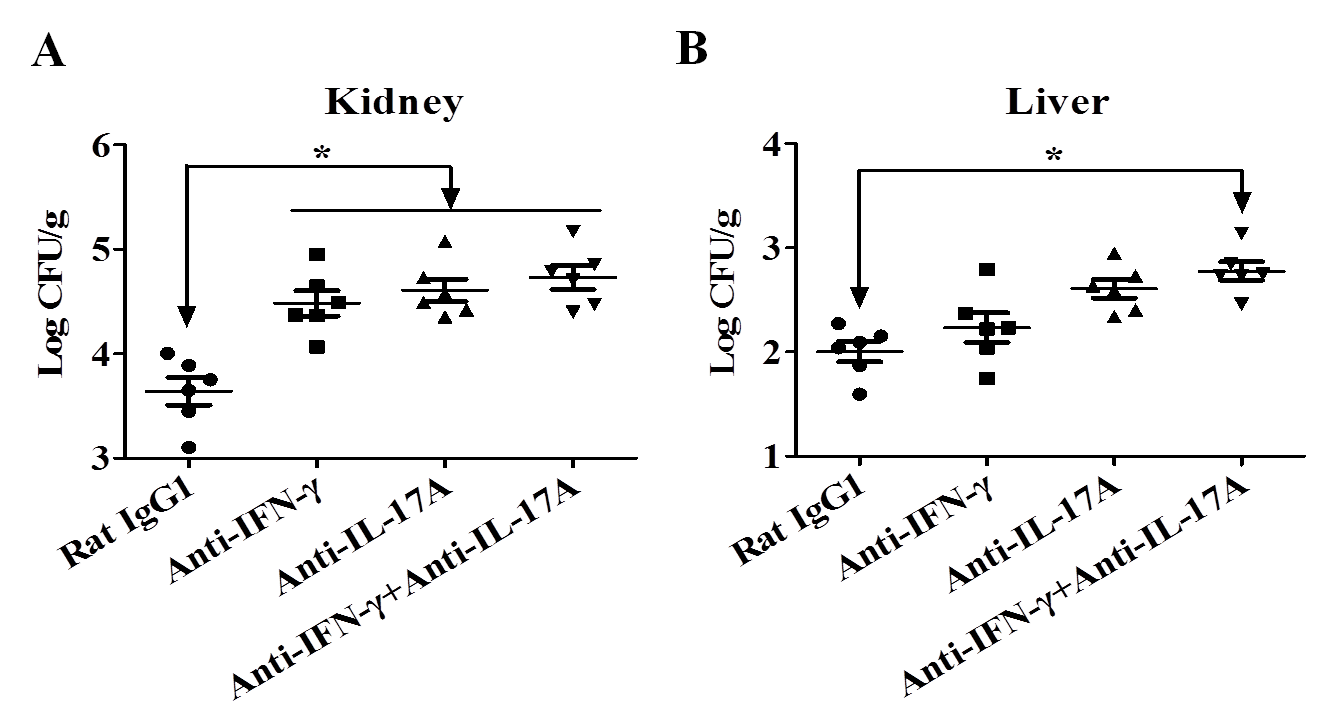

Supplement: S9 Fig — Mice (n = 6 per group) were treated with 500 μg of anti-IFN-γ (clone XMG1.2, BioLegend), 100 μg of anti-IL-17A (clone TC11-18H10.1, BioLegend), mixture of anti-IFN-γ and anti-IL-17A, or rat IgG1 per mouse 1 day before and at day 1 and 3 after injection of mnn10 mutant strain. *, P < 0.05 (Kruskal-Wallis nonparametric One-way ANOVA with Dunns post-test). (TIF) [file ppat.1005617.s009.tif]

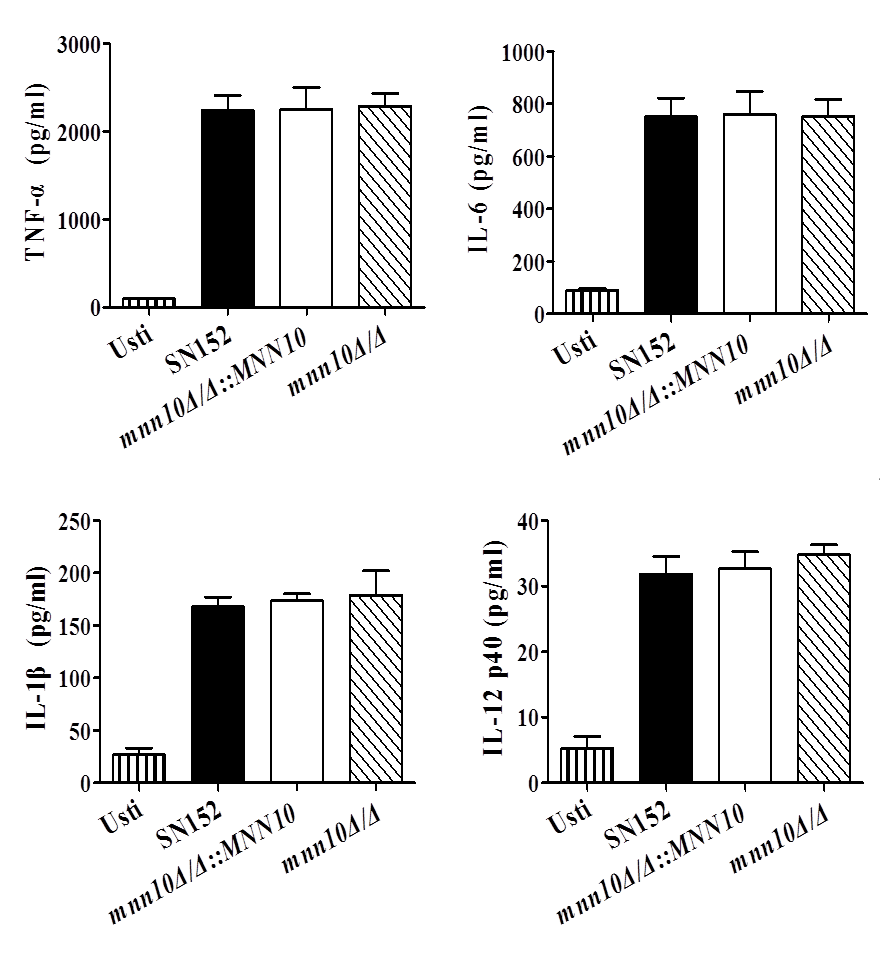

Supplement: S10 Fig — Thioglycollate-elicited peritoneal macrophages were stimulated with the indicated C. albicans hyphae (MOI = 1) for 6 h. Usti, unstimulated. Data are means ± SD of triplicates from one representative experiment of three. (TIF) [file ppat.1005617.s010.tif]

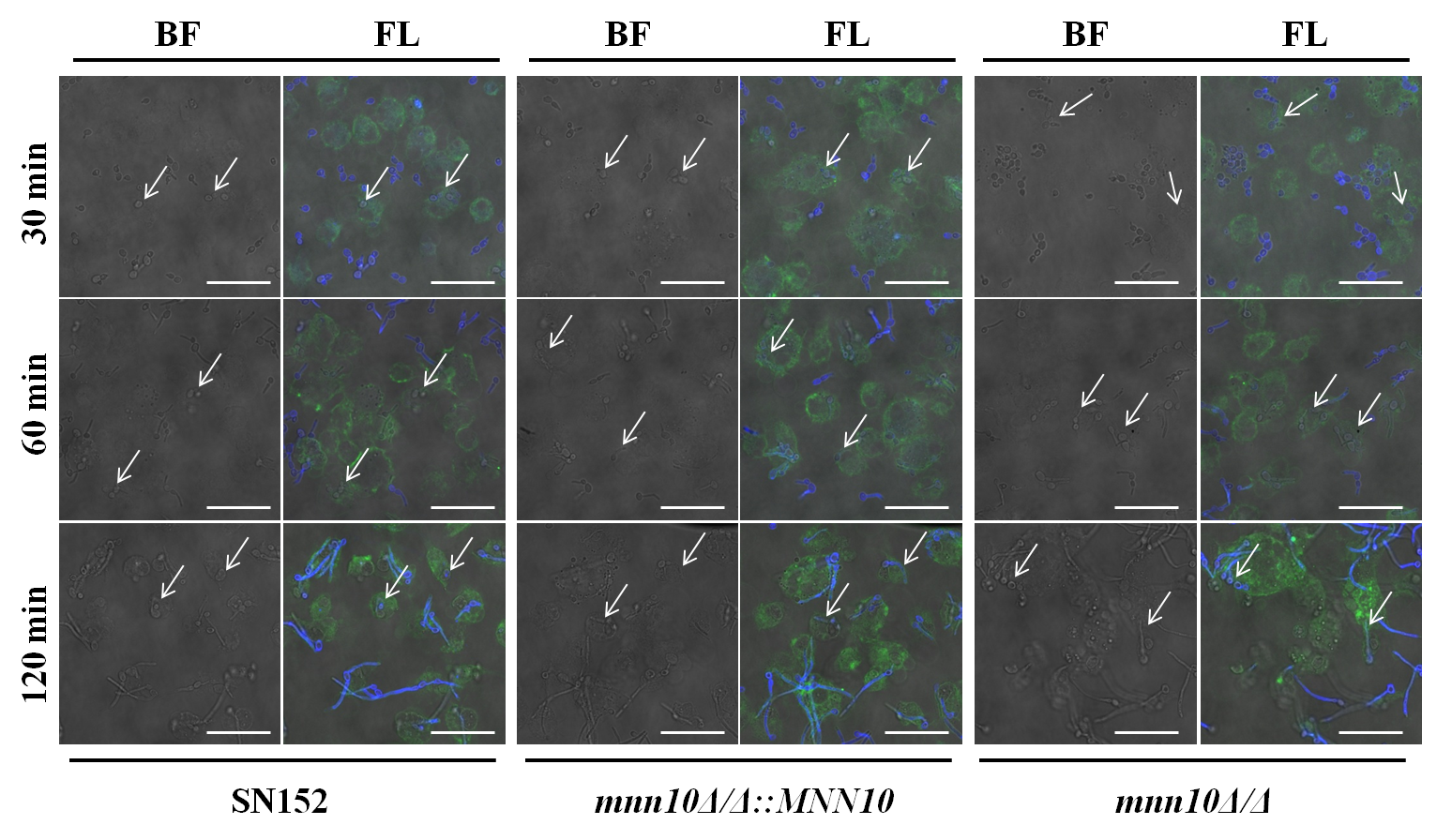

Supplement: S11 Fig — Live C. albicans was added to the macrophages grown on coverslips in multiwell plates at the indicated time. After adding CFW (1 μg/ml) and PSA-FITC (20 μg/ml) to the culture medium for 10 min, the samples were viewed by confocal laser scanning microscope directly. Scale bar represents 10 μm. Arrows indicate the internalized C. albicans cells inaccessible to staining with CFW. Bright field (BF) and fluorescence (FL) are shown individually. (TIF) [file ppat.1005617.s011.tif]

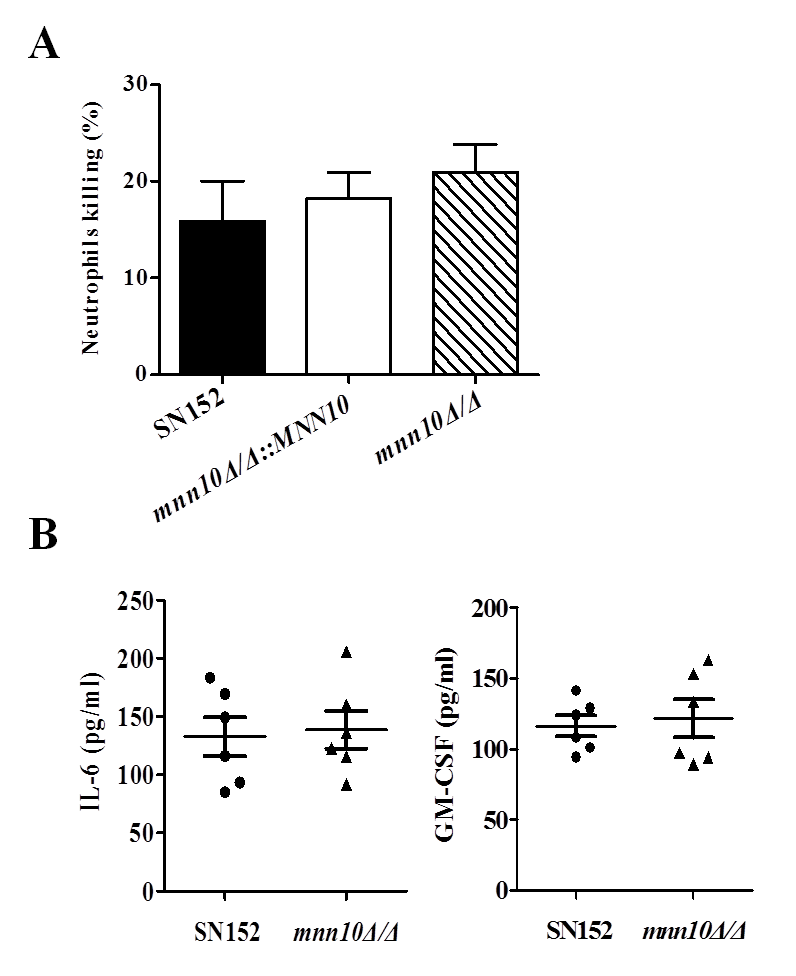

Supplement: S12 Fig — (A) The thioglycollate-elicited peritoneal neutrophils from Dectin-1 deficient mice (6×10^5^ cells) were incubated with 3×10^4^ CFU C. albicans for 1 h. Then the suspension was plated on SDA agar to count live C. albicans colonies. Data are means ± SD of triplicates. (B) ELISA assays for IL-6, GM-CSF in homogenized kidney from infected Dectin-1 deficient mice at day 5 (n = 6 per group). Data are representative of three independent experiments. (TIF) [file ppat.1005617.s012.tif]

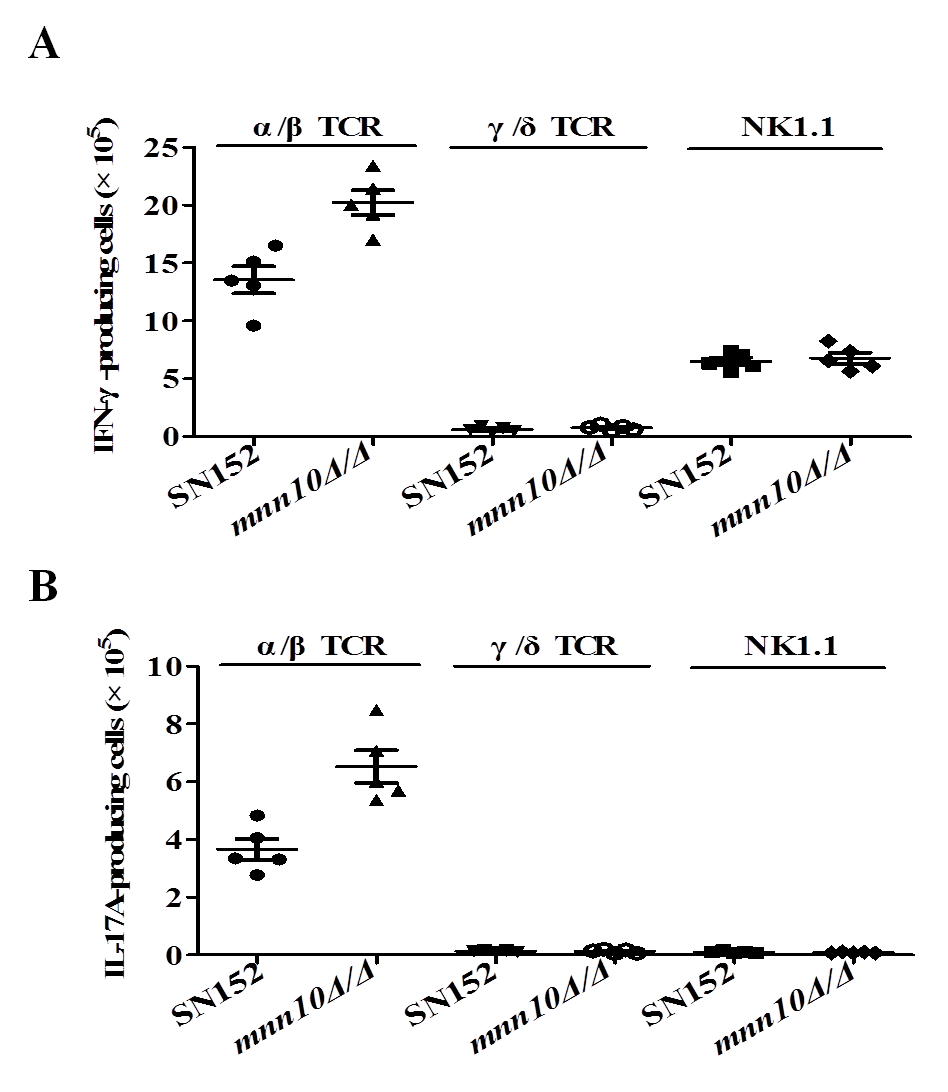

Supplement: S13 Fig — C57BL/6 mice were infected with 5×10^5^ CFU of parental strain SN152 or mnn10 mutant strain via lateral tail vein (n = 5 per group). Intracellular cytokine IFN-γ and IL-17 from α/β or γ/δ T cells were analyzed after gated on CD3+ T cells, and intracellular cytokine signals from NK cells were analyzed after gated on CD3- T cells. (TIF) [file ppat.1005617.s013.tif]
