# Supplementary material for: Mnn10 Maintains Pathogenicity in Candida albicans by Extending α-1,6-Mannose Backbone to Evade Host Dectin-1 Mediated Antifungal Immunity
Source: PLoS Pathog. 2016 May 4;12(5):e1005617. doi: 10.1371/journal.ppat.1005617 (PMC4856274; doi:10.1371/journal.ppat.1005617)
Supplement: S1 Table — (DOCX) [file ppat.1005617.s014.docx]

S1 Table. CWPs identified by LC-MS/MS in *C. albicans*

| Protein | Function | Relative fold change (*mnn10Δ/Δ* versus parental strain ) |
| --- | --- | --- |
| Tdh3p | NAD-linked glyceraldehyde-3-phosphate dehydrogenase | 1.01±0.06 |
| Eno1p | glycolysis and gluconeogenesis; major cell-surface antigen | 0.96±0.05 |
| Met6p | cobalamin-independent methionine synthase | 1.33±0.38 |
| Hsp70p | Putative hsp70 chaperone; heat-shock | 0.86±0.08 |
| Pga4p | β-1,3-glucanosyltransferase; similar to the A. fumigatus GEL family | 0.97±0.32 |
| Utr2p | Putative GPI anchored cell wall glycosidase | 1.03±0.23 |
| Crh11p | GPI-anchored cell wall transglycosylase | 1.04±0.15 |
| Sap9p | Secreted aspartyl protease; roles in adhesion | 1.18±0.04 |
| Pga24p | Similarity to flocculins of *S. cerevisiae* | 1.12±0.37 |
| Ecm33p | GPI-anchored cell wall protein; cell wall biogenesis | 1.04±0.27 |
| Sod1p | Cytosolic copper- and zinc-containing superoxide dismutase | 1.07±0.28 |
| Phr2p | Putative GPI-anchored cell surface glycosidases | 0.79±0.17 |
| Pir32p | Pir-related CWP, putative structural component of the cell wall | 0.89±0.22 |
| Als4p | Putative GPI-anchored protein; Cell-surface adhesin | 0.91±0.21 |
| Dse1p | Essential cell wall protein involved in cell wall integrity and rigidity | 0.83±0.44 |
| Gpm1p | Phosphoglycerate mutase; surface protein that binds host complement factor H and FHL-1 | 1.15±0.44 |
| Dfg5p | *N*-linked mannoprotein of cell wall, GPI modification predicted | 0.11±0.07 |
| Gal102p | UDP-glucose 4,6-dehydratase; role in mannosylation of cell wall proteins | 0.35±0.34 |
| Pmm1p | Phosphomannomutase; enzyme of *O*- and *N*-linked mannosylation; | 0.24±0.11 |
| orf19.5404p | Putative cell wall mannoprotein | 0.43±0.17 |
