# Supplementary material for: Mnn10 Maintains Pathogenicity in Candida albicans by Extending α-1,6-Mannose Backbone to Evade Host Dectin-1 Mediated Antifungal Immunity
Source: PLoS Pathog. 2016 May 4;12(5):e1005617. doi: 10.1371/journal.ppat.1005617 (PMC4856274; doi:10.1371/journal.ppat.1005617)
Supplement: S2 Table — (DOCX) [file ppat.1005617.s015.docx]

S2 Table. Strains used in this study

| Strain | Relevant genotype | Source or reference |
| --- | --- | --- |
| *C. albicans* |  |  |
| SN152 | *arg4Δ/arg4Δ leu2Δ/leu2Δ his1Δ/his1Δ URA3/ura3Δ::imm^434^ IRO1/iro1Δ::imm^434^* | Noble and Johnson (2005) |
| *mnn10Δ/Δ* | *mnn10Δ::HIS1/ mnn10Δ::LEU2 arg4Δ/arg4Δ URA3/ura3Δ::imm^434^ IRO1/iro1Δ::imm^434^* | This study |
| *mnn10Δ/Δ::MNN10* | *mnn10Δ::HIS1/ mnn10Δ::MNN10::SAT1-FLIP arg4Δ/arg4Δ URA3/ura3Δ::imm^434^ IRO1/iro1Δ::imm^434^* | This study |
| *Escherichia coli* |  |  |
| BL21 (DE3) pLysS | F^-^*ompT hsd*SB(rB^-^ mB^-^)*gal* *dcm*(*DE3*)*pLysS*(Cam^R^) | Invitrogen |
| MBP *vec* | BL21 (DE3) pLysS, except for pMAL-p5X | This study |
| MBP*-MNN10* | BL21 (DE3) pLysS, except for pMAL-p5X-*MNN10* | This study |
