# Supplementary material for: Mnn10 Maintains Pathogenicity in Candida albicans by Extending α-1,6-Mannose Backbone to Evade Host Dectin-1 Mediated Antifungal Immunity
Source: PLoS Pathog. 2016 May 4;12(5):e1005617. doi: 10.1371/journal.ppat.1005617 (PMC4856274; doi:10.1371/journal.ppat.1005617)
Supplement: S3 Table — (DOCX) [file ppat.1005617.s016.docx]

S3 Table. Primers used in this study

| Primer | Sequence |
| --- | --- |
| oM1 | GAGGGAAAGCTGGGAAAAAG |
| oM2 | AACCCAAATTGAATCCACCA |
| oM3 | CATGCAATGGGAACACAAAG |
| oM4 | TTCACACCCTGCCATATTCA |
| uB1 | AAATCGGCTCCCGAAACTAT |
| dB2 | ATTTTGTCGGCCATTAGTGC |
| oLY364 | TCAAGCCCTGTAGCTCCATT |
| oLY365 | TCCGCTCATTTGATTTCCTC |
| oLY366 | GCACGCCGTTACAGGAGTTA |
| oLY367 | GAAGTTGGTGACGCGATTGT |
| universal primer 2 | ccgctgctaggcgcgccgtgACCAGTGTGATGGATATCTGC |
| universal primer 5 | gcagggatgcggccgctgacAGCTCGGATCCACTAGTAACG |
| *MNN10* P1 | CATCAGCTGCAAATTGTGGT |
| *MNN10* P3 | cacggcgcgcctagcagcggCTGTGACTGCGGAAAACTCA |
| *MNN10* P4 | gtcagcggccgcatccctgcGGTAGAGATTGCTGGGGTGA |
| *MNN10* P6 | AACCTGTGGAGCTTGTTGCT |
| *MNN10* R1 | ATAAGGGCCCCATCAGCTGCAAATTGTGGT |
| *MNN10* R2 | CCGCTCGAGCTGTGACTGCGGAAAACTCA |
| *MNN10* R3 | CCCACCGCGGATTTCATAACAAAGTACAAG |
| *MNN10* R4 | CCATAGAGCTCAACCTGTGGAGCTTGTTGCT |
| *MNN10* EX1 | CCGGAAGGATTTCAATTTTGGCTGCTAATGAAGG |
| *MNN10* EX2 | CGGAATTCCTACTCCCAAAACTTCCACC |
| *MNN10* RT1 | TGGCTGCTAATGAAGGTGGT |
| *MNN10* RT2 | TTTCCCAGCTTTCCCTCCAC |
